# Humeral shaft fracture: systematic review of non-operative and operative treatment

**DOI:** 10.1007/s00402-023-04836-8

**Published:** 2023-04-24

**Authors:** Saskia H. Van Bergen, Kiran C. Mahabier, Esther M. M. Van Lieshout, Tim Van der Torre, Cornelia A. W. Notenboom, Priscilla A. Jawahier, Michael H. J. Verhofstad, Dennis Den Hartog

**Affiliations:** Trauma Research Unit, Department of Surgery, Erasmus MC, University Medical Center Rotterdam, P.O. Box 2040, 3000 CA Rotterdam, The Netherlands

**Keywords:** Complication, Fracture healing, Humeral shaft fracture, Non-operative treatment, Operative treatment, Review

## Abstract

**Introduction:**

Humeral shaft fractures can be treated non-operatively or operatively. The optimal management is subject to debate. The aim was to compare non-operative and operative treatment of a humeral shaft fracture in terms of fracture healing, complications, and functional outcome.

**Methods:**

Databases of Embase, Medline ALL, Web-of-Science Core Collection, and the Cochrane Central Register of Controlled Trials (CENTRAL) were systematically searched for publications reporting clinical and functional outcomes of humeral shaft fractures after non-operative treatment with a functional brace or operative treatment by intramedullary nailing (IMN; antegrade or retrograde) or plate osteosynthesis (open plating or minimally invasive). A pooled analysis of the results was performed using MedCalc.

**Results:**

A total of 173 studies, describing 11,868 patients, were included. The fracture healing rate for the non-operative group was 89% (95% confidence interval (CI) 84–92%), 94% (95% CI 92–95%) for the IMN group and 96% (95% CI 95–97%) for the plating group. The rate of secondary radial nerve palsies was 1% in patients treated non-operatively, 3% in the IMN, and 6% in the plating group. Intraoperative complications and implant failures occurred more frequently in the IMN group than in the plating group. The DASH score was the lowest (7/100; 95% CI 1–13) in the minimally invasive plate osteosynthesis group. The Constant–Murley and UCLA shoulder score were the highest [93/100 (95% CI 92–95) and 33/35 (95% CI 32–33), respectively] in the plating group.

**Conclusion:**

This study suggests that even though all treatment modalities result in satisfactory outcomes, operative treatment is associated with the most favorable results. Disregarding secondary radial nerve palsy, specifically plate osteosynthesis seems to result in the highest fracture healing rates, least complications, and best functional outcomes compared with the other treatment modalities.

**Supplementary Information:**

The online version contains supplementary material available at 10.1007/s00402-023-04836-8.

## Introduction

Treatment modalities for humeral shaft fractures have evolved over time. Non-operative treatment has been the preferred method for decades since the healing potential of the humerus was considered very good in terms of speed and fracture healing rates, restoration of anatomy is not a prerequisite for good functional outcome, and patients are not exposed to operative risks such as iatrogenic radial nerve palsy, postoperative infections, and implant failure. However, the very good results from functional bracing as published in landmark papers in the 70’s and 80’s by, e.g., Sarmiento, could not be reproduced by others [1]. Despite the possibility of early mobilization of the shoulder and elbow joints, impairment of range of motion (ROM) of especially the shoulder joint was often reported [2, 3]. The persisting clinical need led to the development of new and improved implants for surgical treatment.

Operative treatment for humeral shaft fractures is mostly performed using intramedullary nailing (IMN) or plate osteosynthesis. An IMN is placed in the medullary cavity of the humerus and is, thus, in line with its mechanical axis. If closed reduction can be achieved, periosteal blood supply and fracture biology can be preserved. Incisions are small and require less soft tissue stripping than open reduction and plate osteosynthesis [4]. However, shoulder-related complaints, such as pain and restriction of shoulder movement due to malrotation and impingement of the proximal nail tip or locking head screw, are frequently reported [5–8]. Open reduction and plate osteosynthesis (ORPO) offers the possibility of anatomic reduction and, depending on the fracture configuration, compression of fragments, as it requires extensive soft tissue exposure [9]. A potential disadvantage is a possible higher rate of (temporary) secondary radial nerve palsy [10]. Minimally invasive plate osteosynthesis (MIPO) has the advantage of limited soft tissue dissection and avoids the need to expose the radial nerve [10].

The development of anatomical and angular locked plate systems since approximately 2002 has led to a variety of reliable surgical techniques and a shift from non-operative management toward osteosynthesis, even when no absolute indication for surgery is present [11–15]. Some authors recommend to use IMN, whereas recently MIPO has been proposed as the preferred treatment [6, 8, 16–22]. The debate on the most optimal treatment strategy of humeral shaft fractures remained inconclusive after previous reviews, which only included 6–17 published randomized controlled trials and comparative prospective cohort studies in total [8, 16–26].

The primary aim of the current systematic review and pooled analysis was to compare fracture healing between non-operative and operative treatment of a humeral shaft fracture. The secondary aims were to compare complications and functional outcome.

## Materials and methods

This systematic literature review and pooled analysis was conducted and reported according to the standards set out in Preferred Reporting Items for Systematic Reviews and Meta-Analyses (PRISMA) [27]. Methods used for the analysis, search strategy, and inclusion criteria were specified in advance.

### Search strategy

Databases of Embase, Medline ALL, Web-of-Science Core Collection, and the Cochrane Central Register of Controlled Trials (CENTRAL) were searched. Search strings were made by an experienced librarian and are shown in Table 1. The final search was done on July 30, 2021.

**Table 1:** Search strategy

Database searched	Via	Query	Records	Records after duplicates removed
Embase	Embase.com	((('humerus fracture'/de OR 'humerus shaft fracture'/de OR 'forearm fracture'/de) NOT (proximal OR distal):ab,ti,kw) OR (((humeral-shaft* OR humerus-shaft* OR forearm-shaft* OR arm-shaft*) NEAR/3 (fracture*))):ab,ti,kw) AND (surgery/exp OR surgery:lnk OR 'orthopedic fixation device'/exp OR 'bone plate'/de OR 'conservative treatment'/exp OR brace/de OR 'plaster cast'/de OR splinting/de OR immobilization/exp OR (surg* OR operat* OR nailing OR nails OR pins OR plate* OR plating OR (extern* NEAR/3 fix*) OR screw* OR conservative* OR brace* OR bracing OR sling* OR plaster* OR cast OR casting OR nonoperat* OR nonsurg* OR Sarmiento OR splint* OR traction OR immobili*):ab,ti,kw) NOT ((animal/exp OR animal*:de OR nonhuman/de) NOT ('human'/exp)) NOT ([Conference Abstract]/lim) NOT ('child'/exp NOT ('adult'/exp OR 'adolescent'/de))	5809	5769
Medline ALL	Ovid	((("Humeral Fractures"/) NOT (proximal OR distal).ab,ti,kf.) OR (((humer* OR forearm OR arm) ADJ3 shaft* ADJ3 fracture*)).ab,ti,kf.) AND (surgery.xs. OR exp "Orthopedic Fixation Devices"/ OR braces/ OR immobilization/ OR (surg* OR operat* OR nailing OR nails OR pins OR plate* OR plating OR (extern* ADJ3 fix*) OR screw* OR conservative* OR brace* OR bracing OR sling* OR plaster* OR cast OR casting OR nonoperat* OR nonsurg* OR Sarmiento OR splint* OR traction OR immobili*).ab,ti,kf.) NOT (exp Animals/ NOT Humans/) NOT (news OR congres* OR abstract* OR book* OR chapter* OR dissertation abstract*).pt. NOT ((exp Child/ OR exp Infant/) NOT (exp Adult/ OR exp Adolescent/))	2975	861
Web of Science Core Collection	Web of Knowledge	TS = ((((humer* OR forearm OR arm) NEAR/3 shaft* NEAR/3 fracture*)) AND ((surg* OR operat* OR nailing OR nails OR pins OR plate* OR plating OR (extern* NEAR/3 fix*) OR screw* OR conservative* OR brace* OR bracing OR sling* OR plaster* OR cast OR casting OR nonoperat* OR nonsurg* OR Sarmiento OR splint* OR traction OR immobili*)) NOT ((child* OR infan* OR pediatric*) NOT (adult* OR elderly* OR geriatric*)) NOT ((animal* OR rat OR rats OR mouse OR mice OR murine OR dog OR dogs OR canine OR cat OR cats OR feline OR rabbit OR cow OR cows OR bovine OR rodent* OR sheep OR ovine OR pig OR swine OR porcine OR veterinar* OR chick* OR zebrafish* OR baboon* OR nonhuman* OR primate* OR cattle* OR goose OR geese OR duck OR macaque* OR avian* OR bird* OR fish*) NOT (human* OR patient* OR women OR woman OR men OR man))) AND DT = (Article OR Review OR Letter OR Early Access)	749	91
Cochrane Central Register of Controlled Trials	Wiley	(((humer* OR forearm OR arm) NEAR/3 shaft* NEAR/3 fracture*)):ab,ti,kw AND ((surg* OR operat* OR nailing OR nails OR pins OR plate* OR plating OR (extern* NEAR/3 fix*) OR screw* OR conservative* OR brace* OR bracing OR sling* OR plaster* OR cast OR casting OR nonoperat* OR nonsurg* OR Sarmiento OR splint* OR traction OR immobili*):ab,ti,kw) NOT ((child* OR infan* OR pediatric*) NOT (adult* OR elderly* OR geriatric*)):ab,ti,kw	92	33
Total			9625	6754

Search performed July 30, 2021

### Eligibility criteria

Studies were included if they reported primary treatment of a humeral shaft fracture in patients aged 16 years or older with functional bracing, intramedullary nailing, or plate osteosynthesis. All study designs, except case reports, meta-analyses, and reviews, were included.

Studies were excluded if they met one or more of the following exclusion criteria: (1) recurrent, pathological, or periprosthetic fractures, (2) proximal or distal metaphyseal fracture extension, (3) grade III Gustilo Anderson open fractures, (4) treatment with external fixator, (5) experimental treatment, (6) outcome of less than five patients reported, (7) less than 6 months follow-up, (8) published before the year 2000 or (9) alternative operative methods for humeral shaft fractures (e.g., Ender nails, Marchetti nails, Rushs nails, Hackethal nailing, K wires, expandable, and flexible or elastic nails). Studies that reported on patients with concomitant injuries, such as vascular injury, compartment syndrome, or ipsilateral forearm fractures, were not excluded.

### Study selection

First, four reviewers (KCM, SHVB, TVDT, and CAWN) independently screened the titles and abstracts of the studies to identify eligible studies. Inconsistencies were resolved by consensus. Second, the full-text articles of the remaining eligible publications were retrieved. The corresponding authors of studies with no available full-text version were contacted once by email. Third, the full-text articles were independently reviewed by the aforementioned reviewers. Any disagreement was resolved through consensus. Furthermore, the references of the included studies were reviewed for additional studies that may have been missed.

### Data collection and data items

Data were extracted from the reports independently by three reviewers (KCM, SHVB, and PAJ) using a predefined data sheet. From each study, information was extracted on: study design, publication characteristics, demographics, treatment characteristics (including type of treatment, antegrade or retrograde IMN, ORPO, or MIPO), fracture classification according to the AO/OTA classification, complications, range of motion, and functional outcome scores, including patients-reported outcome measures (PROMs).

Fracture healing (time) was defined as (time to) radiologic or clinical fracture healing. Nonunion was defined as failure to heal at 6 months post-fracture with no progress toward healing seen on the most recent radiographs. Malunion was defined as fracture healing in an abnormal position. Primary radial nerve palsy was defined as radial nerve palsy as a result of initial trauma. Secondary radial nerve palsy was defined as radial nerve palsy as a result of reposition, during non-operative treatment or surgery. Implant failure was defined as the failure of the medical implant. Intraoperative complications included any deviation from the ideal intraoperative course occurring between skin incision and skin closure. Infection was defined as clinically diagnosed infection of (surgical) wounds as a consequence (of the treatment) of the humeral shaft fracture. Shoulder dysfunction was defined as experiencing pain or limited range of motion of the shoulder. Nail protrusion was defined as migration and subsequent protrusion of the intramedullary nail. Subacromial impingement was defined as irritation of the rotator cuff muscles in the subacromial space. (Sub)cutaneous problems included bursitis, cellulitis, granuloma’s, hypertrophic scarring of the wound, and skin irritation, macerations, or abrasions due to prolonged contact with the brace.

When measurements were done at different time points, the outcomes at the 12 months follow-up were used for calculation. The extracted data were compared, and disagreements were resolved by discussion between the three reviewers. Consensus was reached by discussion.

### Risk of bias assessment

The Methodological Index for Non-Randomized Studies (MINORS) instrument was used to assess methodological quality of the included publications [28]. The MINORS scale yields a maximum score of 24 for comparative cohort studies and a maximum of 16 for non-comparative cohort studies, with a higher score indicating better quality. Studies were scored for the various items by three authors (KCM, SHVB, and PAJ) independently. Any disagreement was resolved by consensus. Funnel plots, for each outcome and per treatment type separately, were used to determine the risk of publication bias.

### Statistical analysis

Data were analyzed using MedCalc Statistical Software (Version 18.2.1; MedCalc Software bvba, Ostend, Belgium; http://www.medcalc.org; 2018). Binary outcomes were transformed using a double arcsine transformation to ensure normal distribution [29]. The transformed rates and 95% confidence intervals were transformed back to prevalence estimates. Forest plots were constructed with 95% confidence intervals. Heterogeneity was quantified with Cochran’s *Q* test and *I*^2^ statistic. For the Cochran's Q test, a *p* value < 0.10 was considered statistically significant. A random effects model was used if the *I*^2^ statistic was > 40%. Otherwise, a fixed-effect model was used. Pooled percentages and means were calculated for binary and continuous variables, respectively, and are reported with their 95% confidence intervals (CI). Results are reported per treatment modality or per subgroup if differences between subgroups were deemed relevant.

## Results

### Study selection

**Fig. 1:**
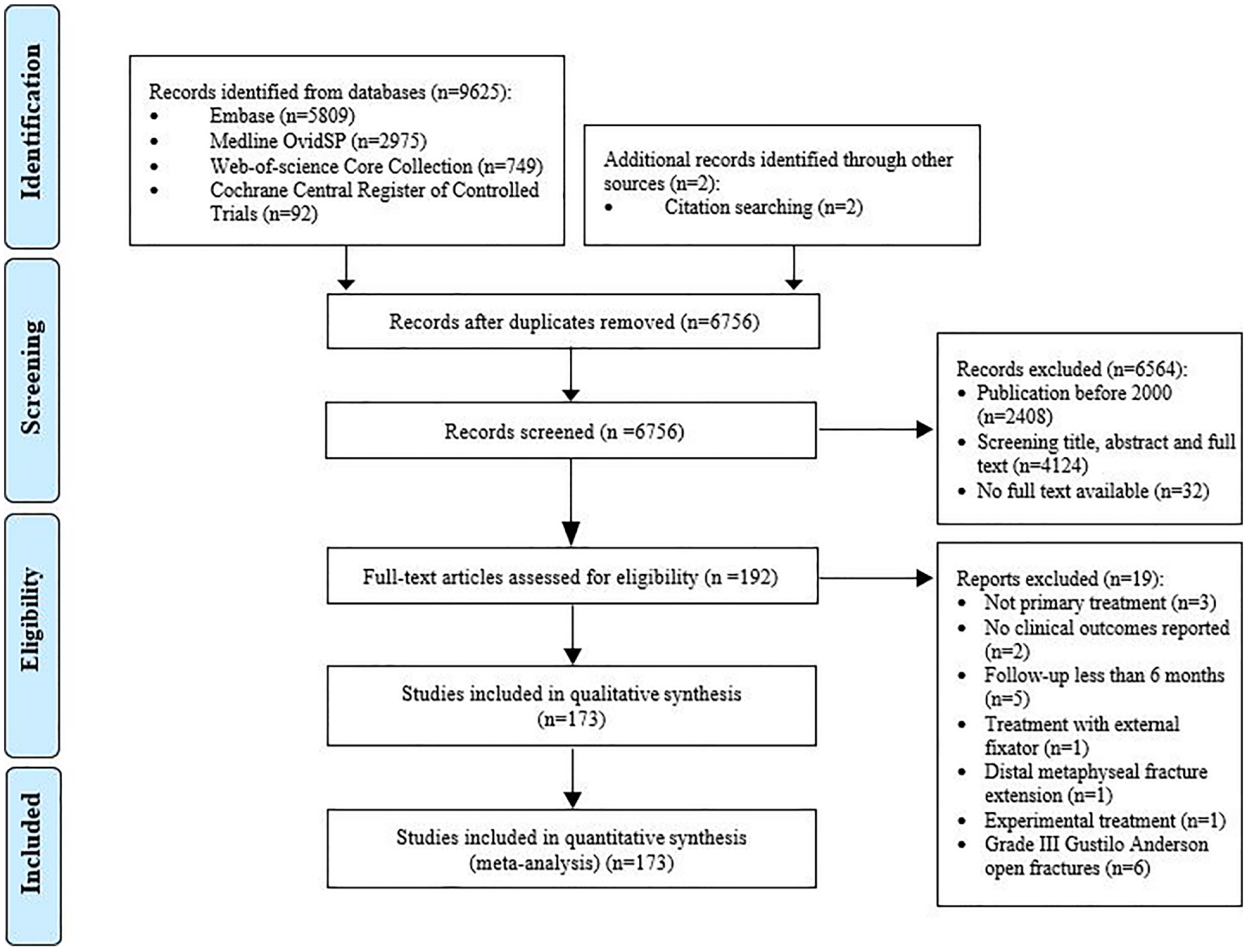
Flow diagram of study selection

The search strings identified 9625 publications (Fig. 1). Duplicates were removed, resulting in 6754 unique publications. Two additional records were identified through other sources (citation searching). The remaining 6756 publications were reviewed for inclusion and exclusion criteria. A total of 192 eligible publications were identified. For 39, studies the full-text manuscripts were not available online. Of these, 13 publications had no contact details available. The remaining corresponding authors were contacted. This revealed seven full-text publications. After full-text assessment, 173 publications were included in this review and meta-analysis (Supplemental Table S1).

### Study characteristics

Supplemental Table S1 shows the study characteristics of all included studies. Of the 173 included studies, 23 were randomized controlled trials, 55 were prospective cohort studies, and 95 were retrospective cohort studies. A total of 79 studies were comparative studies and 94 studies were non-comparative. The included studies report on a total of 11,868 patients. Of these, 2204 were treated non-operatively with a functional brace, 3545 were treated with intramedullary nailing, and 6119 by plate osteosynthesis. The pooled mean age of the patients was 44 years in the non-operative group, 45 in the IMN group, and 41 in the plating group. The pooled mean percentage of males was 57% in the non-operative group, 62% in the IMN group, and 64% in the plate group. The pooled percentage of patients with AO type A fractures was 67% in the non-operative group, 53% in the IMN group, and 46% in the plating group. The pooled percentage of patients with AO type B fractures was 23% in the non-operative group, 34% in the IMN group, and 36% in the plating group. The pooled percentage of patients with AO type C fractures was 9% in the non-operative group, 12% in the IMN group, and 15% in the plating group.

### Risk of bias assessment

The outcome of the methodological quality assessment, according to the MINORS score, is shown in Supplemental Table S2. The average score of the quality assessment for comparative studies was 20/24 (range 11–23) and 12/16 points (range 9–15) for non-comparative studies.

### Fracture healing—time to union

Time to fracture healing (radiologic or clinical) was reported in 37 studies (Table 2). The pooled estimate time to fracture healing was 16 weeks (95% CI 14–18 weeks) for the non-operative group, 14 weeks (95% CI 13–15 weeks) for the IMN group, and 15 weeks (95% CI 14–16 weeks) for the plate group. An antegrade IMN approach resulted in a pooled estimate time to fracture healing of 14 weeks (95% CI 12–15 weeks) versus 12 weeks (95% CI 9–16 weeks) after a retrograde approach. Furthermore, considering plate osteosynthesis, ORPO resulted in a pooled estimate time to fracture healing of 16 weeks (95% CI 15–17 weeks) versus 14 weeks (95% CI 12–16 weeks) after MIPO. Much heterogeneity of effects was seen across studies in all treatment groups, varying from 91% in the MIPO group to 98% in the (antegrade) IMN group.

**Table 2:** Fracture healing of a humeral shaft fracture per treatment group

	Treatment	Study arms	Population	Cases	Heterogeneity		Pooled value
		*N*	*N*	*N*	Cochran’s *Q* (*p* value)	*I*^2^ (%) (95% CI)	(95% CI)
Fracture healing time^a^ (weeks) [7, 10, 13, 57, 58, 60, 66, 70, 96, 106, 107, 112, 118, 119, 121, 126, 129, 131, 132, 134, 135, 144, 150, 151, 160, 162, 163, 166, 171, 176, 177, 181–184, 186, 192]	Non-operative	5	286	N.A	60 (< 0.001)	93 (87–97)	16.4 (14.4–18.4)
	IMN	21	819	N.A	977 (< 0.001)	98 (98–98)	13.8 (12.5–15.1)
	Antegrade	17	654	N.A	777 (< 0.001)	98 (97–98)	13.8 (12.4–15.2)
	Retrograde	3	87	N.A	56 (< 0.001)	96 (92–98)	12.4 (9.1–15.8)
	Plate	41	1392	N.A	1555 (< 0.001)	97 (97–98)	15.4 (14.4–16.4)
	ORPO	31	1194	N.A	1416 (< 0.001)	98 (98–98)	15.8 (14.7–17.0)
	MIPO	10	198	N.A	101 (< 0.001)	91 (86–94)	14.1 (12.2–15.9)
Fracture healing^b^ (%) [1, 3, 5, 7, 10, 11, 13, 22, 30–76, 79–85, 87–97, 99, 100, 102–107, 110–120, 122–139, 141–150, 152–186, 188–194]	Non-operative	26	1979	1770	193 (< 0.001)	87 (82–91)	89 (84–92)
	IMN	73	2990	2811	156 (< 0.001)	54 (40–65)	94 (92–95)
	Antegrade	55	2195	2060	88 (< 0.001)	39 (15–56)	94 (92–95)
	Retrograde	8	265	255	9 (0.221)	26 (0–67)	94 (91–97)
	Plate	136	5226	5030	227 (< 0.001)	41 (27–52)	96 (95–97)
	ORPO	91	3896	3728	171 (< 0.001)	47 (33–59)	96 (95–96)
	MIPO	45	1330	1302	46 (0.394)	4 (0–31)	98 (97–98)
Nonunion^c^ (%) [1, 5, 7, 10, 22, 30–33, 35–39, 41–50, 53–60, 62–76, 79–90, 92–95, 97, 99, 100, 102–106, 110–114, 116–120, 122–139, 141–150, 152–194]	Non-operative	24	1959	182	175 (< 0.001)	87 (82–91)	11 (7–15)
	IMN	70	2787	156	106 (< 0.001)	35 (12–51)	6 (5–7)
	Antegrade	55	2181	127	80 (0.013)	32 (5–51)	6 (5–8)
	Retrograde	7	238	10	7 (0.278)	20 (0–64)	5 (2–8)
	Plate	129	5098	163	205 (< 0.001)	37 (22–50)	3 (3–4)
	ORPO	88	3865	139	167 (< 0.001)	48 (33–60)	4 (3–5)
	MIPO	41	1233	24	33 (0.764)	0 (0–23)	2 (2–3)
Malunion^d^ (%) [5, 30, 31, 33, 43, 49, 50, 53, 57, 64, 69–71, 76, 79–81, 85–87, 91, 92, 94, 97, 99, 103–107, 110, 114, 115, 132, 138, 143, 144, 146, 148–151, 157, 160, 161, 163, 166, 168, 171, 176, 178, 180, 182, 183, 185, 188, 191–194]	Non-operative	11	486	34	48 (< 0.001)	79 (63–88)	6 (2–12)
	IMN	22	798	23	53 (< 0.001)	61 (37–75)	3 (1–5)
	Antegrade	17	555	20	50 (< 0.001)	68 (47–81)	3 (1–6)
	Retrograde	1	N.A	N.A	N.A	N.A	0 (0–4)
	Plate	59	1939	15	29 (1.000)	0 (0–0)	1 (1–2)
	ORPO	37	1293	6	11 (1.000)	0 (0–0)	1 (1–2)
	MIPO	22	646	9	15 (0.805)	0 (0–26)	2 (1–3)

95% CI 95% confidence interval, IMN intramedullary nailing, MIPO minimally invasive plate osteosynthesis, N.A. not applicable, ORPO open reduction plate osteosynthesis

^a^Fracture healing time was defined as time to radiologic or clinical fracture healing

^b^Fracture healing was defined as radiologic or clinical fracture healing

^c^Nonunion was defined as failure to heal at 6 months post-fracture with no progress towards healing seen on the most recent radiographs

^d^Malunion was defined as fracture healing in an abnormal position

### Fracture healing rate

In 160/173 (92%) studies consisting of 10,206 patients the fracture healing rate was reported (Table 2). The pooled fracture healing rate for the non-operative group was 89% (95% CI 84–92%), 94% (95% CI 92–95%) for the IMN group, and 96% (95% CI 95–97%) for the plating group. The pooled fracture healing rate was the highest in the MIPO group (98%; 95% CI 97–98%). In the non-operative group, high heterogeneity across studies was found (*I*^2^ = 87%) and seen in the funnel plot (Supplemental Fig. S1). In the IMN and plate group, the funnel plots showed comparable asymmetry and the heterogeneity was moderate (*I*^2^ = 54% and *I*^2^ = 41%, respectively; Supplemental Figure S1).

### Fracture healing—nonunion

The pooled nonunion rate showed variation between the treatment groups (Table 2). In the non-operative group, 182 nonunions were reported in 1959 patients, resulting in a pooled estimate of 11% (95% CI 7–15%). In the IMN group, 156 nonunions were reported in 2787 patients, resulting in a pooled estimate of 6% (95% CI 5–7%) and in the plating group, 163 nonunions were reported in 5098 patients, resulting in a pooled estimate of 3% (95% CI 3–4%). In the plating group, an open approach resulted in more nonunions than a minimally invasive approach [4% (95% CI 3–5%) and 2% (95% CI 2–3%), respectively].

### Fracture healing—malunion

Pooled malunion rates were 6% (95% CI 2–12%) in the non-operatively treated group, 3% (95% CI 1–5%) in the IMN group, and 1% (95% CI 1–2%) in the plating group (Table 2). However, malunion was often poorly defined and is expected to be reported differently across studies.

### Complications—radial nerve palsy

The pooled primary radial nerve palsy rate showed no variation between the treatment groups (Table 3). Secondary radial palsy was reported in 146 studies (Table 3). The pooled secondary radial nerve palsy rate was 1% (95% CI 0–2%, 18 studies, *N* = 1377, 10 patients) in the non-operatively treated group, 3% (95% CI 2–3%, 58 studies, *N* = 2576, 66 patients) in the IMN group, 4% (95% CI 3–5%, 42 studies, *N* = 1292, 43 patients) in the MIPO group, and 7% (95% CI 6–9%, 82 studies, *N* = 4232, 275 patients) in the ORPO group.

**Table 3:** Complication rates of (treatment of) a humeral shaft fracture per treatment group

	Treatment	Study arms	Population	Cases		Heterogeneity		Pooled value	
		*N*	*N*		*N*	Cochran’s *Q* (*p* value)	*I*^2^ (%) (95% CI)		(%) (95% CI)
Primary radial nerve palsy^a^ [1, 5, 7, 13, 22, 30, 33–36, 38–42, 44–49, 53–58, 60, 63, 65, 67, 69, 71, 73–75, 77, 80, 81, 83–85, 88, 89, 92–95, 98, 102–104, 106, 110, 112, 115, 116, 118, 119, 126, 131–136, 139–142, 145, 148, 149, 156, 157, 159, 160, 162, 166–170, 172–174, 176, 178–188, 190–194]	Non-operative	23	1739		142	24 (0.364)	7 (0–40)		8 (7–10)
	IMN	44	1933		116	172 (< 0.001)	75 (67–81)		6 (4–8)
	Antegrade	31	1255		74	148 (< 0.001)	80 (72–85)		5 (3–8)
	Retrograde	6	213		11	8 (0.166)	36 (0–75)		5 (3–9)
	Plate	85	3371		291	494 (< 0.001)	83 (79–86)		6 (4–8)
	ORPO	62	2603		29	398 (< 0.001)	85 (81–88)		7 (4–9)
	MIPO	23	768		262	65 (< 0.001)	66 (48–78)		4 (2–7)
Secondary radial nerve palsy^b^ [1, 5, 7, 10, 11, 13, 22, 30–43, 46–51, 53–56, 58–61, 63–71, 73, 75, 76, 78–85, 87–100, 102–108, 110–115, 117–123, 125–136, 139, 142–145, 147–152, 155–158, 160–162, 165–176, 178–188, 190–194]	Non-operative	19	1377		10	36 (0.001)	50 (15–71)		1 (0–2)
	IMN	62	2576		66	81 (0.044)	25 (0–45)		3 (2–3)
	Antegrade	47	1872		39	57 (0.121)	20 (0–45)		2 (2–3)
	Retrograde	7	224		8	3 (0.751)	0 (0–50)		4 (2–8)
	Plate	136	5524		318	348 (< 0.001)	61 (53–68)		6 (5–7)
	ORPO	92	4232		275	287 (< 0.001)	68 (61–74)		7 (6–9)
	MIPO	44	1292		43	49 (0.250)	12 (0–40)		4 (3–5)
Intraoperative complications^c^ [7, 22, 30, 31, 38, 39, 43, 58–60, 62, 63, 66, 67, 71, 73, 78, 80, 83–85, 90, 94, 97, 99, 104, 108, 112, 113, 119, 131, 135, 136, 139, 149, 150, 158, 162, 165, 167, 174, 176, 178–186, 188, 193, 194]	Non-operative	N.A	N.A		N.A	N.A	N.A		N.A
	IMN	40	1489		59	180 (< 0.001)	78 (71–84)		5 (3–8)
	Antegrade	27	872		33	62 (< 0.001)	58 (35–73)		4 (2–6)
	Retrograde	6	202		10	13 (< 0.001)	63 (9–85)		5 (1–11)
	Plate	43	1868		6	25 (0.980)	0 (0–0)		1 (0–1)
	ORPO	29	1409		4	18 (0.933)	0 (0–7)		1 (0–1)
	MIPO	14	459		2	7 (0.897)	0 (0–18)		1 (0–2)
Implant failure^d^ [5, 7, 10, 11, 31–33, 41, 42, 46, 48, 49, 54–56, 58–60, 62–64, 67, 68, 71, 73, 75, 76, 78, 83, 84, 86, 88, 90–92, 96, 97, 104, 105, 109, 113, 118, 119, 121, 122, 125, 132, 134, 135, 150, 152, 161, 162, 167–169, 176, 178–180, 182–186, 188, 193, 194]	Non-operative	N.A	N.A		N.A	N.A	N.A		N.A
	IMN	28	1034		51	48 (< 0.001)	44 (12–64)		4 (3–6)
	Antegrade	20	668		31	41 (< 0.001)	54 (23–72)		4 (2–6)
	Retrograde	3	128		8	3 (0.232)	32 (0–98)		7 (3–12)
	Plate	71	2839		40	88 (0.076)	20 (0–41)		2 (1–2)
	ORPO	50	2300		31	67 (0.043)	27 (0–49)		2 (1–3)
	MIPO	21	539		9	19 (0.515)	0 (0–45)		2 (1–4)
Infection^e^ [5, 7, 10, 22, 30–32, 37–39, 41, 43, 46, 48–50, 52, 54–56, 58, 60, 64, 67–73, 76–88, 90–93, 96, 97, 99, 100, 102–106, 108, 110–119, 121–123, 125–127, 129, 131, 133–135, 137, 139, 142–150, 152, 153, 155, 156, 158, 160–162, 165–176, 178–188, 190–194]	Non-operative	9	462		3	6 (0.685)	0 (0–51)		1 (0–2)
	IMN	60	2416		34	71 (0.143)	16 (0–40)		2 (1–2)
	Antegrade	48	1863		28	63 (0.059)	25 (0–48)		2 (1–2)
	Retrograde	6	235		2	5 (0.431)	0 (0–75)		1 (0–3)
	Plate	117	5108		124	193 (< 0.001)	40 (25–52)		3 (2–3)
	ORPO	83	3982		116	158 (< 0.001)	48 (33–60)		3 (3–4)
	MIPO	34	1126		8	22 (0.934)	0 (0–7)		1 (1–2)

95% CI 95% confidence interval, IMN intramedullary nailing, MIPO minimally invasive plate osteosynthesis, N.A. not applicable, ORPO open reduction plate osteosynthesis

^a^Primary radial nerve palsy was defined as radial nerve palsy as a result of initial trauma

^b^Secondary radial nerve palsy was defined as radial nerve palsy as a result of reposition, during non-operative treatment or surgery

^c^Intraoperative complications were defined as any deviation from the ideal intraoperative course occurring between skin incision and skin closure

^d^Implant failure was defined as the failure of the medical implant

^e^Infection was defined as clinically diagnosed infection of (surgical) wounds as a consequence (of the treatment) of the humeral shaft fracture

### Complications—intraoperative complications

The pooled rate of intraoperative complications was 5% (95% CI 3–8%) in patients treated with an IMN and 1% (95% CI 0–1%) in patients treated with plate osteosynthesis (Table 3). Heterogeneity across studies was especially low in the plate group (*I*^2^ = 0%).

### Complications—implant-related complications

Implant failures were reported more frequently in the IMN group (51/1034, pooled estimate of 4%; 95% CI 3–6%) than in patients in the plate group [pooled estimate of 2% (95% CI 1–2%), 40/2839 patients; Table 3]. An antegrade IMN approach resulted in less implant failures than a retrograde approach [4% (95% CI 3–6%) and 7% (95% CI 3–12%), respectively]. Implant failure did not differ between the surgical approaches in the plating group [ORPO 2% (95% CI 1–3%) and MIPO 2% (95% CI 1–4%)].

### Complications—infection

The infection rate was reported in 124 studies consisting of 7986 patients, and was low in all treatment groups, especially in the non-operative [1% (95% CI 0–2%), 3/462 patients] and MIPO group [1% (95% CI 1–2%), 8/1126 patients; Table 3]. The infection rate in the IMN and ORPO group was 2% (95% CI 1–2%) and 3% (95% CI 3–4%), respectively.

### Complications—shoulder dysfunction

The pooled rate of shoulder dysfunction was the highest in patients treated with an IMN (11%; 95% CI 8–15%) and the lowest in patients treated with plate osteosynthesis (6% (95% CI 4–8%); Supplemental Table S3). An antegrade IMN resulted in more shoulder dysfunction than a retrograde IMN [13% (95% CI 10–16%) and 5% (95% CI 1–15%), respectively].

### Complications—nail protrusion

The pooled rate of nail protrusion was 10% (95% CI 6–14%) in patients treated with an IMN (17 studies, 61/666 patients; Supplemental Table S3).

### Complications—subacromial impingement

Subacromial impingement was seen more in the antegrade IMN group than in the plate osteosynthesis group [pooled rate of 13% (95% CI 9–18%) and 2% (95% CI 1–3%), respectively; Supplemental Table S3].

### Complications—(sub)cutaneous problems

The pooled rate of (sub)cutaneous problems in patients treated non-operatively was 6% (95% CI 4–9%, nine studies, 20/347 patients; Supplemental Table S3).

### Range of motion

In the plating group, the pooled estimates of shoulder abduction and anteflexion were 151° (95% CI 116–186°) and 148° (95% CI 137–160°), respectively (Table 4). Anteflexion was better after MIPO than after ORPO [167° (95% CI 164–171°) and 141° (95% CI 124–158°), respectively]. In the IMN group, consisting of only 2 studies with a total of 34 patients, the pooled estimates of shoulder abduction and anteflexion were 132° (95% CI 76–189°) and 120° (95% CI 33–207°), respectively. All treatment groups showed high heterogeneity across studies, varying from 87% in the MIPO group to 100% in all other operative treatment groups.

**Table 4:** Range of motion after treatment of a humeral shaft fracture per treatment group

		Study arms	Population	Heterogeneity		Pooled value
		*N*	*N*	Cochran’s *Q* (*p* value)	*I*^2^ (%) (95% CI)	(degrees) (95% CI)
Shoulder abduction [76, 79, 115, 125, 153]	Non-operative	0	N.A	N.A	N.A	N.A
	IMN	2	34	309 (< 0.001)	100 (99–100)	132 (76–189)
	Antegrade	2	34	309 (< 0.001)	100 (99–100)	132 (76–189)
	Retrograde	0	N.A	N.A	N.A	N.A
	Plate	9	194	25,064 (< 0.001)	100 (100–100)	151 (116–186)
	ORPO	8	146	19,692 (< 0.001)	100 (100–100)	148 (111–186)
	MIPO	1	N.A	N.A	N.A	N.A
Shoulder anteflexion [10, 76, 79, 96, 107, 115, 153]	Non-operative	0	N.A	N.A	N.A	N.A
	IMN	2	34	181 (< 0.001)	100 (99–100)	120 (33–207)
	Antegrade	2	34	181 (< 0.001)	100 (99–100)	120 (33–207)
	Retrograde	0	N.A	N.A	N.A	N.A
	Plate	14	289	5444 (< 0.001)	100 (100–100)	148 (137–160)
	ORPO	10	181	5202 (< 0.001)	100 (100–100)	141 (124–158)
	MIPO	4	108	24 (< 0.001)	87 (70–95)	167 (164–171)

95% CI 95% confidence interval, IMN intramedullary nailing, MIPO minimally invasive plate osteosynthesis, N.A. not applicable, ORPO open reduction plate osteosynthesis

### Functional outcome—DASH

The DASH score after on average 1 year (ranging from 6 to 24 months) showed variation in mean scores between the treatment groups (Table 5). For the non-operative group, the pooled estimate score was 17/100 (95% CI 3–31); for the IMN group, it was 23/100 (95% CI 17–29); and for the plating group, it was 13/100 (95% CI 8–19; Table 4). The DASH score was the highest in the antegrade IMN group (23/100; 95% CI 17–29) and the lowest in the MIPO group (7/100; 95% CI 1–13).

**Table 5:** Functional outcome scores after treatment of a humeral shaft fracture per treatment group

Instrument	Treatment	Study arms	Population	Heterogeneity		Pooled value
		***N***	***N***	Cochran’s *Q* (*p* value)	*I*^2^ (%) (95% CI)	(points) (95% CI)
DASH score^a^ [88, 115, 125, 134, 153, 161, 166, 168, 171, 173, 176, 182, 186, 188, 191]	Non-operative	3	114	141 (< 0.001)	99 (98–99)	17 (3–31)
	IMN	5	192	181 (< 0.001)	98 (97–99)	23 (17–29)
	Antegrade	5	192	181 (< 0.001)	98 (97–99)	23 (17–29)
	Retrograde	0	N.A	N.A	N.A	N.A
	Plate	13	378	1292 (< 0.001)	99 (99–99)	13 (8–19)
	ORPO	9	280	936 (< 0.001)	99 (99–99)	17 (9–24)
	MIPO	4	98	97 (< 0.001)	97 (94–98)	7 (1–13)
Constant–Murley score^b^ [7, 11, 62, 66, 79, 110, 125, 128, 143, 153, 158, 161, 172, 176]	Non-operative	1	N.A	N.A	N.A	N.A
	IMN	9	499	2071 (< 0.001)	100 (100–100)	90 (85–95)
	Antegrade	7	440	375 (< 0.001)	98 (98–99)	89 (85–93)
	Retrograde	2	23	N.A	N.A	N.A
	Plate	13	569	199 (< 0.001)	94 (91–96)	93 (92–95)
	Open	10	295	147 (< 0.001)	94 (91–96)	93 (91–95)
	MIPO	3	274	48 (< 0.001)	96 (91–98)	93 (89–97)
UCLA shoulder score^c^ [10, 107, 114, 115, 118, 127, 131, 160, 173]	Non-operative	0	N.A	N.A	N.A	N.A
	IMN	2	49	17 (< 0.001)	94 (81–98)	28 (22–34)
	Antegrade	2	49	17 (< 0.001)	94 (81–98)	28 (22–34)
	Retrograde	0	N.A	N.A	N.A	N.A
	Plate	15	501	385 (< 0.001)	96 (95–97)	33 (32–33)
	Open	8	346	311 (< 0.001)	98 (97–98)	32 (32–33)
	MIPO	7	155	69 (< 0.001)	91 (85–95)	33 (32–34)

95% CI 95% confidence interval, DASH Disabilities of the Arm, Ahoulder and Hand, IMN intramedullary nailing, MIPO minimally invasive plate osteosynthesis, N.A. not applicable, ORPO open reduction plate osteosynthesis, UCLA University of California at Los Angeles

^a^The Disabilities of the Arm, Shoulder, and Hand (DASH) score ranges from 0 to100 points, with a lower score representing less disability [196, 197]

^b^The Constant–Murley score ranges from 0 to 100 points, with a higher score representing better outcome [198]

^c^The University of California at Los Angeles (UCLA) shoulder score ranges from 0 to 35 points, with a higher score representing better outcome [199]

### Functional outcome—Constant–Murley

The pooled estimate of the Constant–Murley score was 90/100 (95% CI 85–95) in the IMN group and 93/100 (95% CI 92–95) in the plating group (Table 5). The Constant–Murley score did not differ between the surgical approaches in the treatment groups.

### Functional outcome—UCLA

The pooled estimate of the UCLA shoulder score in the IMN group was 28/35 (95% CI 22–34) and 33/35 (95% CI 32–33) in the plating group (Table 5). The UCLA shoulder score did not differ between the surgical techniques in the treatment groups.

### Functional outcome—other

Little to no differences were observed in the other functional outcome scores after IMN or plating osteosynthesis (Supplemental Table S4). Heterogeneity was high (*I*^2^ > 70%) in all subgroups for all functional outcomes, most likely due to the low number of studies with available data. For the non-operatively treated patients, little to no data of functional outcome scores were available for analyses.

The Broberg–Morrey, Gill, Hospital for Special Surgery, l’Insalata, Neer Shoulder, Oxford Shoulder Score, *Quick-*DASH, Rommens, Simple Shoulder Test, Short Musculoskeletal Functional Assessment, and Short Form-36, as well as the Hunter criteria did not have enough data reported for analyses. The nowadays seldom used Rodriguez–Merchan criteria were analyzed but not reported.

## Discussion

This systematic review compared fracture healing, complications, and functional outcome of non-operative and operative treatment for humeral shaft fractures and results suggest that although all treatment modalities result in satisfactory outcomes, operative treatment, and specifically plate osteosynthesis, should be considered the preferred treatment as it results in the most favorable fracture healing rates, least complications, and best functional outcomes.

The current systematic review reveals that the risk to develop a nonunion after non-operative treatment is much higher (11%) than after any kind of surgical stabilization (6% and 3% in the IMN and plating group, respectively). This is in line with previous systematic reviews reporting higher absolute risks of nonunion after non-operative treatment (15% and 18%) and a risk ratio of 0.49 for nonunion in the operative group compared with in the non-operative group [8, 24, 25]. A first requirement for good functional recovery is fracture stability since it relieves pain in the upper limb. Stability can be achieved by fracture union, but also by relative or absolute surgical stabilization of a fresh fracture with IMN and plate osteosynthesis, respectively. A nonunion after non-operative treatment implicates that the patient has experienced pain and loss of function for months, whereas a patient who has been operated upon immediately after his injury has been able to recover functionally despite the development of the nonunion. In the balance of shared decision-making, such numbers call for a surgical and not a non-operative treatment.

The final goal of any type of treatment should be a good functional outcome. Overall, all treatment modalities result in satisfactory functional outcomes after 1 year, indicating that a good functional outcome can be achieved irrespective of treatment. However, a slight advantage of functional recovery can be found after operative treatment with plate osteosynthesis considering the Constant–Murley, DASH, and UCLA shoulder score. This is in line with a meta-analysis of RCTs describing better functional outcomes in patients treated with plate osteosynthesis than in patients treated with IMN [26]. Less complications and rotator cuff problems might enable these patients treated with plate osteosynthesis to regain function faster. These favorable results of functional recovery may tip the scale of the scientific debate toward plate osteosynthesis as the preferred treatment.

However, speed of functional recovery and a lower risk of nonunion after a humeral shaft fracture comes at a price. Both non-operative and operative treatment generate complications. The major complication is considered a radial nerve palsy. Primary nerve palsies are caused by the trauma itself, not by the therapy given to treat the injury. Secondary radial nerve palsy occurs from fracture reduction during non-operative treatment or manipulation during surgery. Not surprisingly, the rate of radial nerve palsy after non-operative treatment is much lower—albeit not absent—than after surgery in which the nerve is exposed. Within the operative group, the current systematic review showed a higher rate of secondary radial nerve palsy in the patients treated with (open) plating. However, the rate of persistent radial nerve palsy could not be defined due to the heterogeneity in reporting, and therefore questions about permanent disability after radial nerve palsy cannot be addressed. A meta-analysis of RCTs and observational studies, comparing non-operative and operative treatment, reported no difference in permanent (primary or secondary) radial nerve palsy rate between both groups suggesting that the risk of persistent radial nerve palsy should no longer be a deterrent for operative treatment [8]. Other complications inherent to operative treatment were more frequently reported in the IMN group than in the plating group. Results of other reviews are comparable, describing lower number of complications in the plating group than in the IMN group, suggesting plating is superior to IMN [18, 21, 26].

All previous meta-analyses only included randomized control trials and comparative prospective cohort studies of 6–17 published studies in total [8, 16–26]. A strength of the current study is that by including many study designs, it included all relevant recent comparative and non-comparative studies, resulting in 173 included studies reporting the results of 11,868 patients. In this way, this systematic review provides information on results of all relevant aspects of each treatment option, and therefore empowers both the patient and the doctor in their respective roles in the desired shared decision-making process.

However, some limitations of this study are the low methodological quality of the included studies as reflected by the MINORS scores. The studies meeting the inclusion criteria often had small sample sizes and lacked an adequate power calculation. Unfortunately, due to the lack of homogeneous reporting of, e.g., patient characteristics and treatment regimens of functional bracing, risk factor and subgroup analyses could not be performed. Furthermore, different outcome parameters and methods of reporting the results were used. Results were frequently reported without a standard deviation and thus could not be included in the pooled analysis. Therefore, the results of this study should be interpreted with care given the large statistical and clinical heterogeneity.

In the literature, a definitive answer on the optimal treatment strategy remains as high-quality data are lacking. This causes practice variation. Furthermore, uniform reporting of outcome of treatment is needed to compare the results of different studies. For instance, in the included studies, 18 different functional outcome scores were reported. The use of different instruments makes it hard to compare results. The DASH and Constant–Murley score have been validated and are recommended as preferred instruments for future studies [195].

## Conclusion

This study suggests that even though all treatment modalities result in satisfactory outcomes, operative treatment is associated with the most favorable results. Disregarding secondary radial nerve palsy, specifically plate osteosynthesis seems to result in the highest fracture healing rates, least complications, and best functional outcomes compared with the other treatment modalities.

## Supplementary Information

Below is the link to the electronic supplementary material.

Supplementary file1 (JPG 395 KB)

Supplementary file2 (JPG 637 KB)

Supplementary file3 (DOCX 80 KB)

Supplementary file4 (DOCX 41 KB)

Supplementary file5 (DOCX 25 KB)

Supplementary file6 (DOCX 24 KB)

## Data Availability

No additional data are available. Data can be made available upon reasonable request to the principal investigator.
