# Supplementary figures and images for: Humeral shaft fracture: systematic review of non-operative and operative treatment
Source: Arch Orthop Trauma Surg. 2023 Apr 24;143(8):5035–54. doi: 10.1007/s00402-023-04836-8 (PMC10374687; doi:10.1007/s00402-023-04836-8)

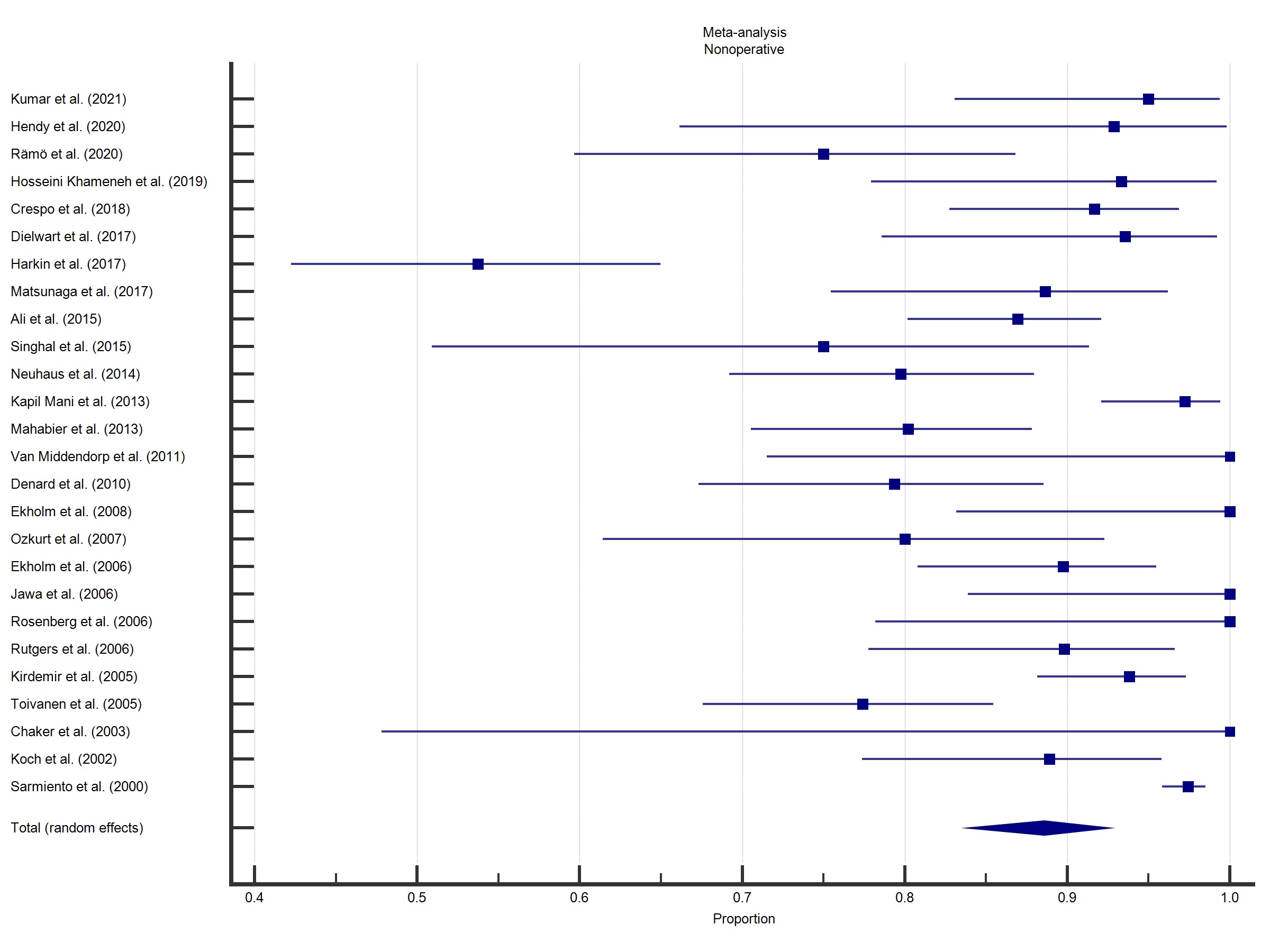

Supplement: Supplementary file 1 — Supplementary file1 (JPG 395 KB) [file 402_2023_4836_MOESM1_ESM.jpg]

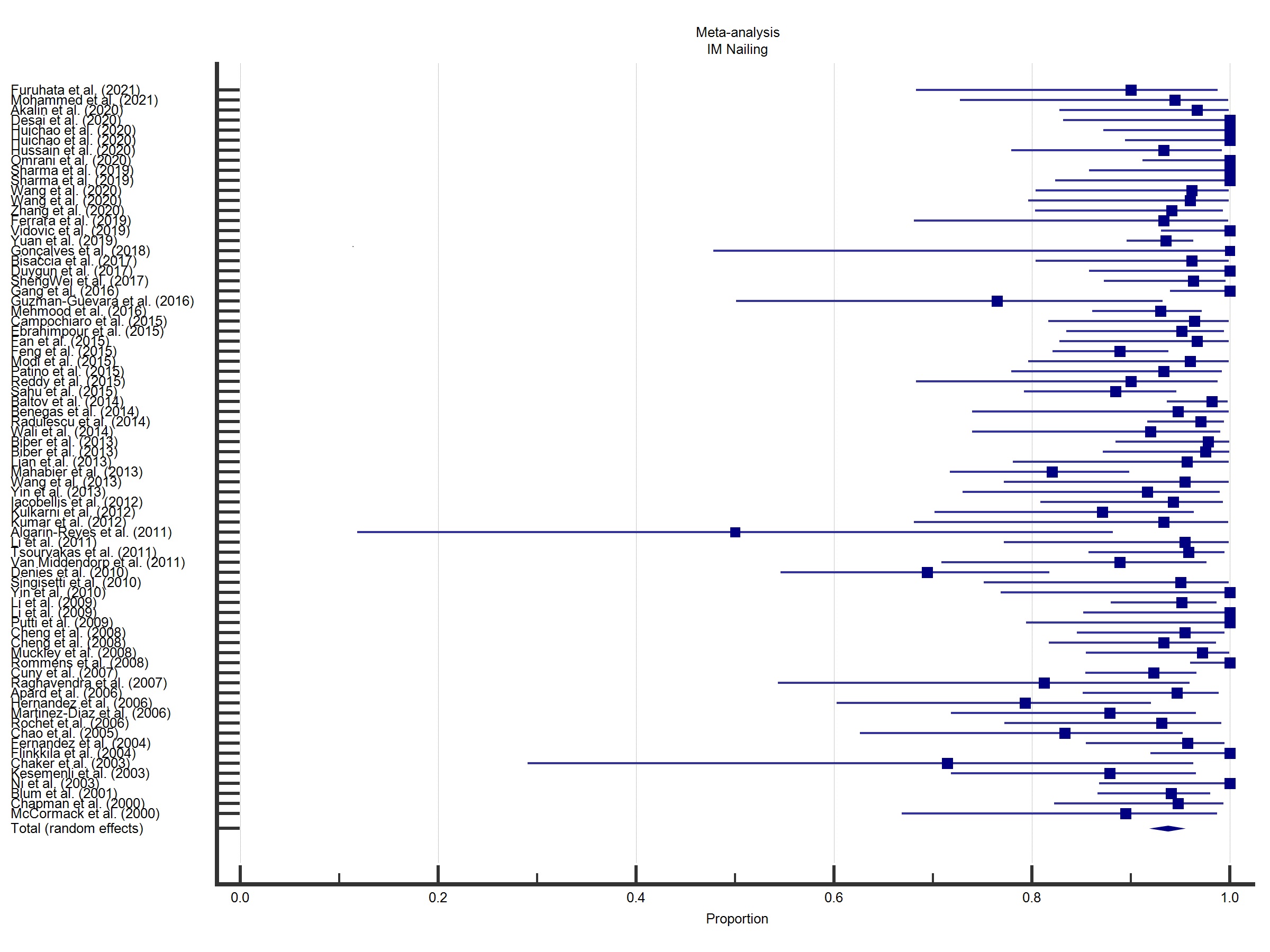

Supplement: Supplementary file 2 — Supplementary file2 (JPG 637 KB) [file 402_2023_4836_MOESM2_ESM.jpg]
