# Supplementary material for: Humeral shaft fracture: systematic review of non-operative and operative treatment
Source: Arch Orthop Trauma Surg. 2023 Apr 24;143(8):5035–54. doi: 10.1007/s00402-023-04836-8 (PMC10374687; doi:10.1007/s00402-023-04836-8)
Supplement: Supplementary file 3 — Supplementary file3 (DOCX 80 KB) [file 402_2023_4836_MOESM3_ESM.docx]

**Supplemental Table S1. Study characteristics**

|  | **Study design** | **Single or multicenter** | **Treatment per group** | **N** | **Age**  (mean (range)) | **Males**  N (%) | **Follow-up**  **per group**  (mean) (range)) | **OTA/AO class**  N (%) | | |  |
| --- | --- | --- | --- | --- | --- | --- | --- | --- | --- | --- | --- |
|  |  |  |  |  | (years) |  | (months) | **A** | **B** | **C** | |
| Sarmiento *et al.* 2000 [1] | Prospective | Single center | Nonoperative | 620 | 36 (16-83) | 391 (63%) | N.R. | N.R. | N.R. | N.R. | |
| Chapman *et al.* 2000 [30] | RCT | Single center | Antegrade IMN | 38 | 33 (18-70) | 26 (68%) | 15 (4-48) | N.R. | N.R. | N.R. | |
|  |  |  | ORPO | 46 | 34 (18-83) | 25 (54%) | 12 (4-48) | N.R. | N.R. | N.R. | |
| McCormack *et al.* 2000 [31] | RCT | Multicenter | Antegrade and retrograde IMN | 21 | 40 (19-82) | 13 (62%) | 14 (6-33) | 3 (48%) | 11 (38%) | 5 (14%) | |
|  |  |  | ORPO | 23 | 49 (20-81) | 15 (65%) | 14 (6-33) | 8 (35%) | 10 (48%) | 4 (17%) | |
| Paris *et al.* 2000 [32] | Retrospective | Single center | ORPO | 156 | 45 (16-96) | 95 (61%) | N.R. | N.R. | N.R. | N.R. | |
| Tingstad *et al.* 2000 [33] | Retrospective | Single center | ORPO | 82 | 33 (13-79) | 44 (54%) | N.R. | N.R. | N.R. | N.R. | |
| Blum *et al.* 2001 [34] | Prospective | Multicenter | Antegrade and retrograde IMN | 84 | 56 (17-91) | 46 (55%) | 12 (6-23) | 45 (53%) | 30 (36%) | 9 (11%) | |
| Koch *et al.* 2002 [35] | Retrospective | Single center | Nonoperative (monotrauma) | 54 | 39 (N.R.) | 35 (65%) | N.R. | 32 (59%) | 14 (26%) | 8 (15%) | |
|  |  |  | Nonoperative (polytrauma) | 13 | 23 (N.R.) | N.R. | N.R. | 9 (67%) | 3 (25%) | 1 (8%) | |
| Chaker *et al.* 2003 [36] | Retrospective | Single center | Nonoperative | 5 | 31 (16-59) | 5 (28%) | 38 (18-72) | N.R. | N.R. | N.R. | |
|  |  |  | Retrograde IMN | 7 | 31 (16-59) | 7 (39%) | 38 (18-72) | N.R. | N.R. | N.R. | |
|  |  |  | ORPO | 6 | 31 (16-59) | 6 (33%) | 38 (18-72) | N.R. | N.R. | N.R. | |
| Kesemenli *et al.* 2003 [37] | Prospective | Single center | Antegrade IMN | 33 | 42 (21-61) | 24 (73%) | 42 (28-72) | N.R. | N.R. | N.R. | |
|  |  |  | ORPO | 27 | 33 (19-47) | 19 (70%) | 42 (28-72) | N.R. | N.R. | N.R. | |
| Ni *et al.* 2003 [38] | Retrospective | Single center | Antegrade and retrograde IMN | 26 | N.R. (18-55) | 19 (73%) | 16 (6-24) | 22 (85%) | 3 (12%) | 1 (4%) | |
| Fernandez *et al.* 2004 [39] | Prospective | Single center | Retrograde IMN | 51 | 48 (17-89) | 31 (61%) | 15 (6-44) | 35 (69%) | 15 (29%) | 1 (2%) | |
| Flinkkila *et al.* 2004 [40] | Retrospective | Single center | Antegrade IMN | 44 | 51 (16-75) | 24 (55%) | 66 (24-240) | 30 (68%) | 12 (27%) | 2 (5%) | |
|  |  |  | ORPO | 29 | 41 (17-69) | 18 (62%) | 74 (12-180) | 19 (66%) | 10 (34%) | N.R. | |
| Livani *et al.* 2004 [41] | Prospective | Single center | MIPO | 15 | 36 (14-66) | 11 (73%) | 24 (21-28) | 5 (33%) | 7 (47%) | 3 (20%) | |
| Niall *et al.* 2004 [42] | Retrospective | Single center | ORPO | 49 | 33 (17-72) | 30 (61%) | N.R. | 30 (61%) | 18 (37%) | 1 (2%) | |
| Chao *et al.* 2005 [43] | Retrospective | Single center | Antegrade IMN | 24 | 47 (20-72) | 15 (63%) | 20 (12-44) | N.R. | N.R. | N.R. | |
|  |  |  | ORPO | 36 | 53 (19-85) | 20 (56%) | 92 (12-130) | N.R. | N.R. | N.R. | |
| Kirdemir *et al.* 2005 [44] | Retrospective | Single center | Nonoperative | 129 | 26 (18-58) | 85 (66%) | 13 (3-24) | N.R. | N.R. | N.R. | |
| Toivanen *et al.* 2005 [45] | Retrospective | Single center | Nonoperative | 93 | 53 (16-90) | 38 (41%) | N.R. | 83 (89%) | 10 (11%) | N.R. | |
| Rosenberg *et al.* 2006 [3] | Prospective | Single center | Nonoperative | 15 | 42 (25-71) | 10 (67%) | 30 (12-57) | 9 (60%) | 4 (27%) | 2 (13%) | |
| Apard *et al.* 2006 [46] | Prospective | Single center | Retrograde IMN | 58 | 55 (16-95) | 30 (52%) | 10 (N.R.) | 32 (57%) | 13 (24%) | 11 (19%) | |
| Ekholm *et al.* 2006 [47] | Retrospective | Multicenter | Nonoperative | 78 | 53 (N.R.) | 33 (42%) | N.R. | N.R. | N.R. | N.R. | |
| Hernandez *et al.* 2006 [48] | Retrospective | Single center | Antegrade IMN | 35 | 51 (19-84) | 21 (60%) | 5 (3-6) | 19 (63%) | 10 (37%) | N.R. | |
| Jawa *et al.* 2006 [49] | Retrospective | Single center | Nonoperative | 21 | 41 (24-92) | 9 (43%) | 21 (2-45) | N.R. | N.R. | N.R. | |
| Martinez-Diaz *et al.* 2006 [50] | Retrospective | Single center | Antegrade IMN | 33 | 73 (60-89) | 7 (21%) | 15 (2-48) | 26 (79%) | 5 (15%) | 2 (6%) | |
| Pospula *et al.* 2006 [51] | Retrospective | Single center | MIPO | 12 | 30 (17-46) | 11 (92%) | N.R. | N.R. | N.R. | N.R. | |
| Rochet *et al.* 2006 [52] | Retrospective | Single center | Antegrade IMN | 29 | 42 (17-81) | 18 (62%) | 36 (9-84) | N.R. | N.R. | N.R. | |
| Rutgers *et al.* 2006 [53] | Retrospective | Single center | Nonoperative | 49 | 42 (19-86) | 25 (51%) | 14 (2-50) | N.R. | N.R. | N.R. | |
| Cuny *et al.* 2007 [54] | Retrospective | Single center | Antegrade IMN | 104 | 53 (N.R.) | 62 (42%) | 32 (6-72) | 65 (55%) | 34 (30%) | 5 (15%) | |
| Jiang *et al.* 2007 [55] | Prospective | Single center | MIPO | 21 | 43 (18-75) | 14 (67%) | 29 (19-37) | N.R. | N.R. | 21 (100%) | |
| Numbela *et al.* 2007 [56] | Prospective | Single center | MIPO | 7 | N.R. | N.R. | 12 (N.R.) | N.R. | 5 (72%) | 2 (28%) | |
| Ozkurt *et al.* 2007 [57] | Prospective | Single center | Nonoperative | 30 | 34 (18-64) | 19 (63%) | 20 (10-58) | N.R. | N.R. | N.R. | |
| Raghavendra *et al.* 2007 [58] | Prospective | Single center | Antegrade IMN | 18 | 40 (18-70) | 15 (83%) | N.R. | 11 (61%) | 7 (39%) | N.R. | |
|  |  |  | ORPO | 18 | 41 (22-70) | 17 (94%) | N.R. | 16 (89%) | 2 (11%) | N.R. | |
| Zhiquan *et al.* 2007 [59] | Prospective | Single center | MIPO | 13 | 38 (25-60) | 9 (69%) | 13 (7-19) | 10 (8%) | 1 (77%) | 2 (15%) | |
| Rommens *et al.* 2008 [22] | Retrospective | Single center | Antegrade and retrograde IMN | 99 | 63 (N.R.) | 36 (36%) | 28 (N.R.) | 43 (43%) | 40 (40%) | 16 (16%) | |
| Cheng *et al.* 2008 [60] | RCT | Single center | Antegrade IMN | 44 | 43 (N.R.) | 26 (59%) | 19 (14-26) | 28 (64%) | 13 (30%) | 3 (7%) | |
|  |  |  | Retrograde IMN | 45 | 48 (N.R.) | 28 (62%) | 20 (14-27) | 36 (80%) | 7 (16%) | 2 (5%) | |
| Ekholm *et al.* 2008 [61] | Retrospective | Single center | Nonoperative | 20 | 58 (16-91) | 5 (25%) | 26 (N.R.) | 39 (50%) | 26 (33%) | 13 (17%) | |
| Muckley *et al.* 2008 [62] | Prospective | Single center | Antegrade and retrograde IMN | 36 | 48 (19-83) | 21 (58%) | 22 (6-34) | 13 (36%) | 21 (58%) | 2 (6%) | |
| An *et al.* 2009 [63] | Retrospective | Single center | ORPO | 21 | 39 (24-62) | 13 (62%) | 32 (13-48) | 14 (67%) | 7 (33%) | N.R. | |
|  |  |  | MIPO | 19 | 39 (19-60) | 14 (74%) | 25 (14-44) | 3 (16%) | 13 (68%) | 3 (16%) | |
| Apivatthakakul *et al.* 2009 [64] | Retrospective | Single center | MIPO | 20 | 46 (20-72) | 16 (70%) | 14 (N.R.) | 4 (20%) | 5 (25%) | 11 (55%) | |
| Ji *et al.* 2009 [65] | Retrospective | Single center | MIPO | 22 | N.R. (24-62) | N.R. | 17 (11-14) | N.R. | N.R. | N.R. | |
| Li *et al.* 2009 [66] | Retrospective | Single center | Antegrade IMN | 82 | 38 (N.R.) | 59 (72%) | N.R. | 49 (60%) | 8 (10%) | 25 (31%) | |
|  |  |  | Retrograde IMN | 23 | 34 (N.R.) | 19 (83%) | N.R. | 15 (65%) | N.R. | 8 (35%) | |
| Putti *et al.* 2009 [67] | RCT | Single center | Antegrade IMN | 16 | 36 (23-84) | N.R. | 24 (N.R.) | N.R. | N.R. | N.R. | |
|  |  |  | ORPO | 18 | 39 (22-65) | N.R. | 24 (N.R.) | N.R. | N.R. | N.R. | |
| Wang *et al.* 2009 [68] | Retrospective | Single center | MIPO | 15 | 35 (16-59) | 11 (73%) | 10 (5-18) | 6 (40%) | 8 (53%) | 1 (7%) | |
| An *et al.* 2010 [10] | Retrospective | Single center | ORPO | 16 | 37 (24-62) | 9 (56%) | 33 (13-48) | N.R. | N.R. | N.R. | |
|  |  |  | MIPO | 17 | 38 (19-60) | 12 (71%) | 26 (14-44) | N.R. | N.R. | N.R. | |
| Concha *et al.* 2010 [69] | Prospective | Single center | MIPO | 35 | 33 (19-54) | 26 (74%) | 12 (6-24) | 7 (20%) | 1 (29%) | 18 (51%) | |
| Denard *et al.* 2010 [70] | Retrospective | Multicenter | Nonoperative | 63 | 36 (N.R.) | 34 (54%) | 7 (N.R.) | N.R. | N.R. | N.R. | |
|  |  |  | ORPO | 150 | 35 (N.R.) | 82 (55%) | 8 (N.R.) | N.R. | N.R. | N.R. | |
| Denies *et al.* 2010 [71] | Retrospective | Single center | Antegrade and retrograde IMN | 49 | 52 (N.R.) | 21 (43%) | N.R. | 32 (65%) | 14 (29%) | 3 (6%) | |
|  |  |  | ORPO | 42 | 48 (N.R.) | 25 (60%) | N.R. | 23 (55%) | 15 (36%) | 4 (10%) | |
| Kobayashi *et al.* 2010 [72] | Prospective | Single center | MIPO | 14 | 37 (19-75) | 11 (79%) | 14 (12-21) | 5 (36%) | 9 (64%) | N.R. | |
| Singisetti *et al.* 2010 [73] | Prospective | Single center | Antegrade IMN | 20 | N.R. | N.R. | 12 (10-24) | N.R. | N.R. | N.R. | |
|  |  |  | ORPO | 16 | N.R. | N.R. | 12 (10-24) | N.R. | N.R. | N.R. | |
| Yin *et al.* 2010 [74] | Retrospective | Single center | Antegrade and retrograde IMN | 18 | 36 (20-56) | 14 (78%) | 11 (8-15) | N.R. | N.R. | N.R. | |
| Ziran *et al.* 2010 [75] | Retrospective | Multicenter | MIPO | 32 | N.R. | N.R. | 16 (3-38) | N.R. | N.R. | N.R. | |
| Algarin-Reyes *et al.* 2011 [76] | Retrospective | Single center | Antegrade IMN | 6 | 31 (18-50) | 2 (33%) | 3 (N.R.) | 4 (67%) | 2 (33%) | N.R. | |
|  |  |  | ORPO (anterior approach) | 8 | 31 (8-64) | 4 (50%) | 3 (N.R.) | 2 (25%) | 3 (38%) | 3 (38%) | |
|  |  |  | ORPO (posterior approach) | 9 | 29 (19-46) | 6 (67%) | 3 (N.R.) | 4 (44%) | 3 (33%) | 2 (22%) | |
|  |  |  | ORPO (multiple plates) | 8 | 31 (18-52) | 7 (88%) | 3 (N.R.) | 2 (25%) | 6 (75%) | N.R. | |
| Grass *et al.* 2011 [77] | Retrospective | Single center | Antegrade IMN | 38 | 53 (16-92) | 18 (47%) | 25 (N.R.) | N.R. | N.R. | N.R. | |
| Kirin *et al.* 2011 [78] | Retrospective | Single center | ORPO | 420 | 38 (N.R.) | 340 (81%) | N.R. | N.R. | N.R. | N.R. | |
| Li *et al.* 2011 [79] | RCT | Single center | Antegrade IMN | 22 | 40 (N.R.) | 16 (73%) | 12 (N.R.) | 10 (45%) | 9 (41%) | 3 (14%) | |
|  |  |  | ORPO | 23 | 36 (N.R.) | 16 (70%) | 12 (N.R.) | 5 (22%) | 12 (52%) | 6 (26%) | |
| Lopez-Arevalo *et al.* 2011 [80] | Retrospective | Single center | MIPO | 86 | 44 (14-78) | 60 (70%) | N.R. | 44 (51%) | 34 (40%) | 8 (9%) | |
| Prasarn *et al.* 2011 [81] | Retrospective | Single center | ORPO | 15 | 50 (21-86) | 7 (47%) | 30 (13-37) | 5 (40%) | 4 (33%) | 4 (27%) | |
| Shetty *et al.* 2011 [82] | Prospective | Single center | MIPO | 32 | 39 (22-70) | 19 (59%) | 31 (24-40) | 9 (28%) | 10 (31%) | 13 (41%) | |
| Tsourvakas *et al.* 2011 [83] | Prospective | Single center | Antegrade IMN | 52 | 52 (18-72) | 36 (69%) | 18 (12-24) | 20 (42%) | 15 (31%) | 13 (27%) | |
| Van Middendorp *et al.* 2011 [84] | Prospective | Multicenter | Nonoperative | 14 | 51 (17-84) | 5 (36%) | 12 (N.R.) | 9 (79%) | 2 (21%) | N.R. | |
|  |  |  | Retrograde IMN | 33 | 53 (17-86) | 19 (58%) | 12 (N.R.) | 25 (70%) | 2 (30%) | N.R. | |
| Brunner *et al.* 2012 [85] | Retrospective | Single center | MIPO | 15 | 62 (40-96) | 7 (47%) | 27 (12-38) | 5 (33%) | 7 (47%) | 3 (20%) | |
| Firat *et al.* 2012 [86] | Retrospective | Single center | Nonoperative | 62 | 39 (18-69 | 38 (61%) | 74 (20-132) | 53 (85%) | 7 (11%) | 2 (3%) | |
|  |  |  | Antegrade IMN | 30 | 39 (18-69 | 23 (77%) | 74 (20-132) | 26 (87%) | 4 (13%) | N.R. | |
|  |  |  | ORPO | 36 | 39 (18-69) | 20 (56%) | 74 (20-132) | 28 (78%) | 7 (19%) | 1 (3%) | |
| Iacobellis *et al.* 2012 [87] | Prospective | Single center | Antegrade IMN | 35 | 65 (25-92) | 14 (40%) | 24 (12-38) | 14 (40%) | 11 (31%) | 10 (29%) | |
| Idoine *et al.* 2012 [88] | Retrospective | Single center | ORPO | 96 | 35 (16-80) | 55 (57%) | 58 (12-120) | N.R. | N.R. | N.R. | |
| Kulkarni *et al.* 2012 [89] | Retrospective | Single center | Antegrade IMN | 31 | 39 (20-60) | 25 (81%) | 10 (N.R.) | 16 (52%) | 14 (45%) | 1 (3%) | |
|  |  |  | ORPO | 25 | 39 (22-77) | 19 (76%) | 12 (N.R.) | 17 (60%) | 7 (28%) | N.R. | |
| Kumar *et al.* 2012 [90] | Prospective | Single center | Antegrade IMN | 15 | 46 (17-69) | 8 (53%) | N.R. (16-19) | 10 (67%) | 5 (33%) | N.R. | |
|  |  |  | ORPO | 15 | 45 (17-69) | 10 (67%) | N.R. (16-19) | 14 (93%) | 1 (7%) | N.R. | |
| Malhan *et al.* 2012 [91] | Prospective | Single center | MIPO | 42 | 34 (18-68) | 28 (67%) | 25 (14-35) | 26 (62%) | 16 (38%) | N.R. | |
| Oh *et al.* 2012 [92] | Retrospective | Single center | ORPO | 30 | 42 (17-82) | 16 (53%) | 22 (N.R.) | 15 (50%) | 8 (27%) | 7 (23%) | |
|  |  |  | MIPO | 29 | 40 (16-83) | 16 (55%) | 18 (N.R.) | 11 (38%) | 11 (38%) | 7 (24%) | |
| Pagonis *et al.* 2012 [93] | Retrospective | Single center | ORPO | 158 | 67 (57-87) | 40 (22%) | 163 (84-288) | 107 (62%) | 15 (14%) | 36 (25%) | |
| Shin *et al.* 2012 [94] | Prospective | Single center | MIPO | 21 | 43 (19-75) | 13 (62%) | 17 (13-25) | 11 (52%) | 9 (43%) | 1 (5%) | |
| Tan *et al.* 2012 [95] | Retrospective | Single center | MIPO | 5 | 35 (20-96) | 5 (100%) | 6 (N.R.) | 3 (60%) | 2 (40%) | N.R. | |
| Yang *et al.* 2012 [96] | Prospective | Single center | ORPO | 19 | 37 (20-75) | 15 (79%) | 17 (12-20) | 9 (47%) | 8 (42%) | 2 (11%) | |
| Zhou *et al.* 2012 [97] | Retrospective | Single center | MIPO | 74 | 57 (34-86) | 26 (35%) | 17 (12-24) | 9 (12%) | 45 (61%) | 20 (27%) | |
| Mahabier *et al.* 2013 [13] | Retrospective | Multicenter | Nonoperative | 91 | 49 (N.R.) | 36 (40%) | N.R. | 6 (N.R.) | 4 (N.R.) | 1 (N.R.) | |
|  |  |  | Antegrade and retrograde IMN | 78 | 60 (N.R.) | 32 (41%) | N.R. | 44 (N.R.) | 28 (N.R.) | 6 (N.R.) | |
|  |  |  | ORPO | 11 | 60 (N.R.) | 6 (55%) | N.R. | 43 (N.R.) | 41 (N.R.) | 7 (N.R.) | |
| Aydin *et al.* 2013 [98] | Retrospective | Single center | Nonoperative | 5 | 20 (19-22) | 5 (100%) | N.R. | N.R. | N.R. | N.R. | |
| Biber *et al.* 2013 [99] | Retrospective | Single center | Antegrade IMN | 46 | 57 (N.R.) | 22 (48%) | N.R. | N.R. | N.R. | N.R. | |
|  |  |  | Retrograde IMN | 41 | 41 (N.R.) | 34 (83%) | N.R. | N.R. | N.R. | N.R. | |
| Boschi *et al.* 2013 [100] | Retrospective | Single center | ORPO | 280 | 34 (30-36) | 280 (100%) | 101 (N.R.) | 210 (75%) | 70 (25%) | N.R. | |
| Chen *et al.* 2013 [101] | Retrospective | Single center | Antegrade and retrograde IMN | 310 | 75 (N.R.) | 77 (25%) | 12 (N.R.) | N.R. | N.R. | N.R. | |
|  |  |  | ORPO | 201 | 73 (N.R.) | 42 (21%) | 12 (N.R.) | N.R. | N.R. | N.R. | |
| Kapil Mani *et al.* 2013 [102] | Prospective | Single center | Nonoperative | 108 | 39 (17-77) | 63 (58%) | N.R. | N.R. | N.R. | N.R. | |
| Lee *et al.* 2013 [103] | Prospective | Single center | ORPO | 35 | 38 (14-70) | 26 (74%) | 28 (24-50) | N.R. | N.R. | N.R. | |
| Lee *et al.* 2013 [104] | Retrospective | Single center | MIPO | 28 | 37 (18-77) | 19 (68%) | 21 (14-31) | 7 (24%) | 16 (59%) | 5 (17%) | |
| Lian *et al.* 2013 [105] | RCT | Single center | Antegrade and retrograde IMN | 23 | 38 (17-77) | 16 (70%) | 15 (10-16) | 8 (35%) | 12 (52%) | 3 (13%) | |
|  |  |  | MIPO | 24 | 39 (17-76) | 15 (63%) | 14 (7-20) | 9 (38%) | 9 (38%) | 6 (25%) | |
| Sharaby *et al.* 2013 [106] | Prospective | Single center | ORPO | 22 | 44 (25-67) | 15 (68%) | 25 (17-36) | 12 (55%) | 8 (36%) | 2 (9%) | |
| Shen *et al.* 2013 [107] | Retrospective | Single center | MIPO (DCP) | 26 | 37 (20-60) | 18 (69%) | 32 (13-62) | 5 (19%) | 15 (58%) | 6 (23%) | |
|  |  |  | MIPO (LCP) | 17 | 43 (18-78) | 10 (59%) | 17 (12-18) | 6 (35%) | 5 (30%) | 6 (35%) | |
| Tyllianakis *et al.* 2013 [108] | Retrospective | Single center | Antegrade IMN | 64 | 42 (17-76) | 33 (52%) | 78 (24-120) | 36 (56%) | 22 (34%) | 6 (9%) | |
| Verdano *et al.* 2013 [109] | Retrospective | Single center | Antegrade IMN | 48 | 51 (16-75) | 26 (54%) | 33 (12-61) | N.R. | N.R. | N.R. | |
| Wang *et al.* 2013 [110] | RCT | Single center | Antegrade IMN | 22 | 40 (N.R.) | 16 (73%) | 18 (N.R.) | 10 (45%) | 9 (41%) | 3 (14%) | |
|  |  |  | ORPO | 23 | 36 (N.R.) | 16 (70%) | 18 (N.R.) | 5 (22%) | 12 (52%) | 6 (26%) | |
| Yi *et al.* 2013 [111] | Prospective | Single center | ORPO | 53 | 39 (16-82) | 34 (64%) | 17 (8-45) | 39 (74%) | 14 (26%) | N.R. | |
| Yin *et al.* 2013 [112] | Retrospective | Single center | Antegrade IMN | 24 | 38 (21-70) | 14 (58%) | 17 (12-24) | N.R. | 19 (79%) | 5 (21%) | |
|  |  |  | ORPO | 22 | 40 (25-74) | 12 (55%) | 17 (12-24) | N.R. | 18 (82%) | 4 (18%) | |
| Balam *et al.* 2014 [113] | Prospective | Single center | MIPO | 37 | 28 (19-43) | 33 (89%) | 18 (12-24) | N.R. | N.R. | N.R. | |
| Baltov *et al.* 2014 [5] | Retrospective | Single center | Antegrade and retrograde IMN | 111 | 49 (17-83) | 71 (64%) | 42 (12-72) | 38 (34%) | 46 (41%) | 27 (24%) | |
| Benegas *et al.* 2014 [114] | RCT | Single center | Antegrade IMN | 19 | 38 (N.R.) | 14 (74%) | 12 (N.R.) | 9 (47%) | 4 (21%) | 6 (32%) | |
|  |  |  | MIPO | 21 | 45 (N.R.) | 12 (57%) | 12 (N.R.) | 12 (57%) | 7 (33%) | 2 (10%) | |
| Huri *et al.* 2014 [115] | Retrospective | Single center | ORPO | 14 | 42 (19-66) | 8 (62%) | 18 (13-30) | 7 (50%) | 5 (36%) | 2 (14%) | |
| Neuhaus *et al.* 2014 [116] | Retrospective | Multicenter | Nonoperative | 79 | 48 (18-91) | 46 (58%) | 9 (2-52) | 79 (100%) | N.R. | N.R. | |
| Radulescu *et al.* 2014 [117] | Prospective | Single center | Antegrade IMN | 102 | N.R. | N.R. | N.R. | N.R. | N.R. | N.R. | |
|  |  |  | ORPO | 82 | N.R. | N.R. | N.R. | N.R. | N.R. | N.R. | |
| Singh *et al.* 2014 [118] | Retrospective | Single center | ORPO (DCP) | 102 | 37 (18-65) | 73 (72%) | 12 (N.R.) | 51 (51%) | 40 (39%) | 11 (10%) | |
|  |  |  | ORPO (LCP) | 110 | 38 (22-64) | 75 (68%) | 12 (N.R.) | 55 (50%) | 43 (39%) | 12 (11%) | |
| Wali *et al.* 2014 [119] | RCT | Single center | Antegrade IMN | 25 | 37 (N.R.) | 21 (84%) | N.R. | 16 (64%) | 6 (24%) | 3 (12%) | |
|  |  |  | ORPO | 25 | 38 (N.R.) | 20 (80%) | N.R. | 17 (68%) | 6 (24%) | 2 (8%) | |
| Wang *et al.* 2014 [120] | Retrospective | Single center | MIPO | 17 | 36 (19-51) | 11 (65%) | 17 (13-31) | 1 (6%) | 15 (88%) | 1 (6%) | |
| Yin *et al.* 2014 [121] | Retrospective | Single center | ORPO (lateral approach) | 30 | 38 (22-71) | 16 (53%) | 16 (12-22) | 5 (17%) | 16 (53%) | 9 (30%) | |
|  |  |  | ORPO (posterior approach) | 26 | 38 (20-73) | 14 (54%) | 16 (12-22) | 6 (23%) | 12 (46%) | 8 (31%) | |
| Zogaib *et al.* 2014 [122] | Retrospective | Single center | MIPO | 22 | 34 (18-66) | 15 (56%) | 52 (6-126) | N.R. | N.R. | N.R. | |
| Zogbi *et al.* 2014 [123] | Retrospective | Single center | MIPO | 7 | 30 (N.R.) | 5 (71%) | 30 (N.R.) | N.R. | N.R. | N.R. | |
| Chen *et al.* 2015 [11] | Retrospective | Single center | ORPO | 43 | 72 (52-88) | 15 (35%) | 37 (18-48) | 5 (14%) | 11 (28%) | 23 (58%) | |
| Ali *et al.* 2015 [124] | Retrospective | Single center | Nonoperative | 156 | 54 (18-92) | 71 (46%) | N.R. | N.R. | N.R. | N.R. | |
| Campochiaro *et al.* 2015 [125] | Retrospective | Single center | Antegrade IMN | 28 | 68 (20-97) | 9 (32%) | 19 (6-32) | N.R. | 25 (89%) | 3 (11%) | |
| Ebrahimpour *et al.* 2015 [126] | Prospective | Single center | Antegrade IMN | 41 | 39 (21-59) | 34 (83%) | 12 (10-24) | N.R. | N.R. | N.R. | |
| Esmailiejah *et al.* 2015 [127] | Prospective | Single center | ORPO | 33 | 35 (16-56) | 8 (25%) | N.R. | 12 (36%) | 10 (30%) | 11 (33%) | |
|  |  |  | MIPO | 32 | 33 (16-53) | 24 (75%) | N.R. | 10 (31%) | 9 (28%) | 13 (41%) | |
| Fan *et al.* 2015 [128] | RCT | Single center | Antegrade IMN | 30 | 39 (N.R.) | 18 (60%) | N.R. | 11 (37%) | 14 (47%) | 5 (17%) | |
|  |  |  | ORPO | 30 | 39 (N.R.) | 19 (63%) | N.R. | 12 (40%) | 15 (50%) | 3 (10%) | |
| Feng *et al.* 2015 [129] | RCT | Single center | Antegrade IMN | 126 | 37 (25-68) | 78 (62%) | N.R. | 59 (47%) | 18 (14%) | 49 (39%) | |
|  |  |  | ORPO | 126 | 38 (24-67) | 69 (55%) | N.R. | 61 (48%) | 23 (18%) | 42 (33%) | |
| Gallucci *et al.* 2015 [130] | Retrospective | Single center | MIPO | 21 | 37 (23-73) | 13 (62%) | 22 (12-50) | N.R. | 20 (95%) | 1 (5%) | |
| Hadhoud *et al.* 2015 [131] | RCT | Single center | ORPO | 15 | 36 (20-65) | 11 (73%) | N.R. (6-10) | 10 (67%) | 4 (27%) | 1 (7%) | |
|  |  |  | MIPO | 15 | 40 (22-67) | 9 (60%) | N.R. (6-10) | 9 (60%) | 3 (20%) | 3 (20%) | |
| Kim *et al.* 2015 [132] | Retrospective | Single center | ORPO | 31 | 26 (18-34) | 31 (100%) | 16 (12-23) | 23 (74%) | 6 (19%) | 2 (7%) | |
| Koca *et al.* 2015 [133] | Retrospective | Single center | ORPO | 11 | 36 (28-50) | 7 (64%) | 14 (6-24) | N.R. | 11 (100%) | N.R. | |
| Kumar *et al.* 2015 [134] | Prospective | Single center | ORPO | 22 | 33 (21-58) | N.R. | 15 (14-17) | 7 (32%) | 13 (59%) | 2 (9%) | |
| Modi *et al.* 2015 [135] | RCT | Single center | Antegrade IMN | 30 | 36 (N.R.) | 20 (80%) | 10 (6-14) | N.R. | N.R. | N.R. | |
|  |  |  | ORPO | 30 | 36 (N.R.) | 17 (74%) | 10 (6-14) | N.R. | N.R. | N.R. | |
| Patino *et al.* 2015 [136] | Retrospective | Single center | Antegrade IMN | 30 | 42 (19-77) | 20 (67%) | 36 (24-60) | 22 (73%) | 7 (23%) | 1 (3%) | |
| Reddy *et al.* 2015 [137] | Prospective | Single center | Antegrade IMN | 20 | N.R. (20-60) | 14 (70%) | N.R. | 10 (50%) | 8 (40%) | 2 (10%) | |
| Sahu *et al.* 2015 [138] | Prospective | Single center | Antegrade IMN | 78 | N.R. | 65 (83%) | N.R. | N.R. | N.R. | N.R. | |
| Sanjeevaiah *et al.* 2015 [139] | Prospective | Single center | MIPO | 42 | 34 (18-68) | 28 (67%) | 25 (14-35) | 26 (62%) | 16 (38%) | N.R. | |
| Shields *et al.* 2015 [140] | Retrospective | Single center | Nonoperative | 32 | 45 (18-84) | N.R. | 47 (12-104) | 20 (63%) | 11 (34%) | 1 (3%) | |
| Singhal *et al.* 2015 [141] | Retrospective | Single center | Nonoperative | 20 | N.R. (16-89) | 10 (50%) | 3 (1-14) | 14 (70%) | 3 (15%) | 3 (15%) | |
| Srinivas *et al.* 2015 [142] | Prospective | Single center | ORPO | 25 | 39 (21-58) | 21 (84%) | N.R. | N.R. | N.R. | N.R. | |
| Wang *et al.* 2015 [143] | Prospective | Single center | ORPO | 23 | 36 (N.R.) | 16 (70%) | N.R. | 5 (22%) | 12 (52%) | 6 (26%) | |
|  |  |  | MIPO | 22 | 39 (N.R.) | 14 (64%) | N.R. | 5 (23%) | 8 (36%) | 9 (41%) | |
| Abril Gaona *et al.* 2016 [144] | Prospective | Single center | ORPO | 19 | 38 (17-78) | 12 (63%) | N.R. | 6 (32%) | 8 (42%) | 5 (26%) | |
| Anand Kumar *et al.* 2016 [145] | Prospective | Single center | ORPO | 54 | N.R. (21-65) | 41 (76%) | 9 (N.R.) | 45 (83%) | 9 (17%) | N.R. | |
| Gang *et al.* 2016 [146] | Retrospective | Single center | Antegrade IMN | 59 | 48 (N.R.) | 31 (53%) | 10 (N.R.) | 22 (37%) | 27 (46%) | 10 (17%) | |
|  |  |  | ORPO | 63 | 51 (N.R.) | 36 (57%) | 10 (N.R.) | 25 (40%) | 32 (51%) | 6 (10%) | |
| Guzmán-Guevara *et al.* 2016 | Prospective | Single center | Antegrade IMN | 17 | 44 (19-81) | N.R. | N.R. | N.R. | N.R. | N.R. | |
| [147] |  |  | ORPO | 23 | 41 (16-79) | N.R. | N.R. | N.R. | N.R. | N.R. | |
| Karunanithi *et al.* 2016 [148] | Prospective | Single center | MIPO | 20 | 41 (21-65) | N.R. | 8 (6-17) | N.R. | N.R. | N.R. | |
| Kumar *et al.* 2016 [149] | Prospective | Single center | ORPO | 59 | N.R. | 41 (69%) | N.R. | N.R. | N.R. | N.R. | |
| Lee *et al.* 2016 [150] | Retrospective | Single center | ORPO | 31 | 41 (23-75) | 11 (41%) | 40 (26-60) | 15 (48%) | 12 (41%) | 4 (11%) | |
| Lee *et al.* 2016 [151] | Retrospective | Single center | ORPO | 28 | 48 (27-82) | 18 (64%) | 26 (12-40) | N.R. | N.R. | N.R. | |
|  |  |  | MIPO | 24 | 51 (16-89) | 15 (63%) | 25 (6-36) | N.R. | N.R. | N.R. | |
| Lu *et al.* 2016 [152] | Retrospective | Single center | ORPO (medial approach) | 16 | 34 (18-56) | 10 (67%) | 12 (N.R.) | 8 (50%) | 5 (31%) | 3 (19%) | |
|  |  |  | ORPO (anterolateral approach) | 18 | 35 (23-59) | 12 (63%) | 12 (N.R.) | 10 (56%) | 4 (22%) | 4 (22%) | |
| Mahajan *et al.* 2016 [153] | Prospective | Single center | MIPO | 57 | N.R. | 36 (75%) | N.R. | 32 (60%) | 15 (25%) | 10 (15%) | |
| Mehmood *et al.* 2016 [154] | RCT | Single center | Antegrade IMN | 100 | 39 (20-59) | 82 (82%) | N.R. | N.R. | N.R. | N.R. | |
|  |  |  | ORPO | 100 | 39 (20-59) | 81 (81%) | N.R. | N.R. | N.R. | N.R. | |
| Wahed *et al.* 2016 [155] | Prospective | Single center | ORPO | 30 | N.R. | N.R. | N.R. | N.R. | N.R. | N.R. | |
| Bisaccia *et al.* 2017 [156] | Prospective | N.R. | Antegrade IMN | 32 | 53 (N.R.) | 15 (47%) | 12 (9-16) | 22 (69%) | 5 (16%) | 5 (16%) | |
|  |  |  | ORPO | 26 | 59 (N.R.) | 11 (42%) | 12 (9-16) | 17 (65%) | 6 (23%) | 3 (12%) | |
| Dielwart *et al.* 2017 [157] | Retrospective | Single center | Nonoperative Brace | 31 | 39 (18-93) | 23 (74%) | 10 (2-34) | 16 (52%) | 7 (23%) | 8 (26%) | |
| Duygun *et al.* 2017 [158] | Prospective | Single center | Antegrade IMN | 24 | 42 (23-55) | 12 (50%) | 24 (12-72) | 21 (88%) | N.R. | 3 (13%) | |
| Harkin *et al.* 2017 [159] | Retrospective | Single center | Nonoperative Brace | 96 | N.R. | 32 (33%) | N.R. | N.R. | N.R. | N.R. | |
| Ko *et al.* 2017 [160] | Retrospective | Single center | ORPO | 23 | 56 (35-67) | 16 (70%) | 19 (N.R.) | N.R. | N.R. | N.R. | |
|  |  |  | MIPO | 27 | 56 (37-70) | 18 (67%) | 19 (N.R.) | N.R. | N.R. | N.R. | |
| Matsunaga *et al.* 2017 [161] | RCT | Single center | Nonoperative Brace | 52 | 40 (N.R.) | 38 (73%) | 12 (N.R.) | 29 (55%) | 17 (33%) | 6 (12%) | |
|  |  |  | ORPO | 58 | 37 (N.R.) | 35 (60%) | 12 (N.R.) | 39 (68%) | 16 (27%) | 3 (5%) | |
| ShengWei *et al.* 2017 [162] | Retrospective | Single center | Antegrade IMN | 54 | 31 (20-41) | 40 (74%) | 15 (6-24) | 28 (52%) | 18 (33%) | 8 (15%) | |
|  |  |  | ORPO | 58 | 31 (18-44) | 38 (67%) | 15 (6-24) | 30 (52%) | 20 (34%) | 8 (14%) | |
| Crespo *et al.* 2018 [163] | Retrospective | Multicenter | Nonoperative Brace | 72 | N.R. | N.R. | 9 (3-13) | N.R. | N.R. | N.R. | |
| Goncalves *et al.* 2018 [164] | Retrospective | Single center | Antegrade IMN | 5 | N.R. | N.R. | N.R. | N.R. | N.R. | N.R. | |
| Ferrara *et al.* 2019 [165] | Retrospective | Single center | Antegrade IMN | 22 | 76 (65-88) | 5 (23%) | N.R. | 8 (36%) | 12 (55%) | 2 (9%) | |
| Hosseini Khameneh *et al.* 2019 | RCT | Single center | Nonoperative Brace | 30 | 49 (19-77) | 26 (87%) | 12 (N.R.) | 21 (70%) | 5 (17%) | 4 (13%) | |
| [166] |  |  | ORPO | 30 | 38 (18-71) | 23 (77%) | 12 (N.R.) | 18 (60%) | 4 (13%) | 8 (27%) | |
| Mehraj *et al.* 2019 [167] | Prospective | Single center | MIPO | 40 | 34 (20-53) | 26 (65%) | 22 (18-28) | N.R. | N.R. | N.R. | |
| Pooja *et al.* 2019 [168] | RCT | Single center | ORPO | 15 | 37 (21-56) | 10 (67%) | 26 (24-31) | 9 (60%) | 4 (27%) | 2 (13%) | |
|  |  |  | MIPO | 15 | 38 (20-56) | 9 (60%) | 27 (25-30) | 10 (67%) | 3 (20%) | 2 (13%) | |
| Seo *et al.* 2019 [169] | Prospective | Single center | ORPO (single plating) | 40 | 44 (N.R.) | 29 (73%) | 14 (N.R.) | 18 (45%) | 16 (40%) | 6 (15%) | |
|  |  |  | ORPO (dual plating) | 20 | 45 (N.R.) | 11 (55%) | 9 (N.R.) | 10 (50%) | 6 (30%) | 4 (20%) | |
| Vidovic *et al.* 2019 [170] | Retrospective | Single center | IMN | 51 | 61 (N.R.) | 20 (39%) | N.R. | 28 (55%) | 14 (27%) | 9 (18%) | |
| Wang *et al.* 2019 [171] | Retrospective | Single center | ORPO | 30 | 43 (19-71) | 17 (57%) | N.R. (6-24) | 8 (27%) | 13 (43%) | 9 (30%) | |
|  |  |  | MIPO | 30 | 38 (21-66) | 12 (40%) | N.R. (6-24) | 7 (23%) | 12 (40%) | 11 (37%) | |
| Yuan *et al.* 2019 [172] | Retrospective | Single center | Antegrade IMN | 204 | 42 (N.R.) | 119 (58%) | 26 (N.R.) | 115 (56%) | 64 (31%) | 25 (12%) | |
|  |  |  | MIPO | 232 | 41 (N.R.) | 128 (55%) | 26 (N.R.) | 127 (55%) | 79 (34%) | 26 (11%) | |
| Li *et al.* 2020 [7] | Retrospective | Single center | ORPO (posterior approach) (A) | 28 | 39 (N.R.) | 22 (79%) | 4 (N.R.) | 28 (100%) | N.R. | N.R. | |
|  |  |  | ORPO (anterolateral approach) (A) | 32 | 38 (N.R.) | 19 (59%) | 4 (N.R.) | 32 (100%) | N.R. | N.R. | |
|  |  |  | ORPO (posterior approach) (B/C) | 29 | 39 (N.R.) | 16 (55%) | 4 (N.R.) | N.R. | 19 (66%) | 10 (35%) | |
|  |  |  | ORPO (anterolateral approach) (B/C) | 18 | 38 (N.R.) | 12 (67%) | 4 (N.R.) | N.R. | 13 (72%) | 5 (28%) | |
| Akalin *et al.* 2020 [173] | RCT | Single center | Antegrade IMN | 33 | 42 (22-88) | 17 (57%) | 24 (N.R.) | 18 (55%) | 9 (27%) | 6 (18%) | |
|  |  |  | ORPO | 33 | 45 (18-88) | 24 (73%) | 24 (N.R.) | 17 (52%) | 11 (33%) | 5 (15%) | |
| Desai *et al.* 2020 [174] | RCT | Single center | Antegrade IMN | 20 | 34 (N.R.) | N.R. | N.R. | N.R. | N.R. | N.R. | |
|  |  |  | ORPO | 20 | 38 (N.R.) | N.R. | N.R. | N.R. | N.R. | N.R. | |
| Hendy *et al.* 2020 [175] | Retrospective | Single center | Nonoperative Brace | 14 | 51 (N.R.) | 7 (50%) | 5 (3-9) | 8 (57%) | 2 (14%) | 4 (29%) | |
| Huichao *et al.* 2020 [176] | Retrospective | Single center | Antegrade IMN | 27 | 48 (N.R.) | 19 (70%) | 14 (N.R.) | 9 (33%) | 14 (52%) | 4 (15%) | |
|  |  |  | Antegrade IMN | 33 | 49 (N.R.) | 22 (67%) | 15 (N.R.) | 12 (36%) | 18 (55%) | 3 (9%) | |
| Hussain *et al.* 2020 [177] | RCT | Single center | Antegrade IMN | 30 | 34 (N.R.) | 24 (80%) | N.R. | N.R. | N.R. | N.R. | |
|  |  |  | ORPO | 30 | 34 (N.R.) | 23 (77%) | N.R. | N.R. | N.R. | N.R. | |
| Omrani *et al.* 2020 [178] | RCT | Single center | Antegrade IMN | 40 | 31 (N.R.) | 25 (63%) | N.R. | N.R. | N.R. | N.R. | |
|  |  |  | ORPO | 40 | 30 (N.R.) | 22 (55%) | N.R. | N.R. | N.R. | N.R. | |
| Rai *et al.* 2020 [179] | Prospective | Single center | ORPO | 150 | N.R. (19-65) | 130 (87%) | N.R. (6-24) | 101 (67%) | 21 (14%) | 28 (19%) | |
| Rämö *et al.* 2020 [180] | RCT | Multicenter | Nonoperative | 44 | 48 (19-80) | 24 (55%) | 12 (N.R.) | 36 (82%) | 7 (16%) | 1 (2%) | |
|  |  |  | ORPO | 38 | 50 (19-81) | 20 (53%) | 12 (N.R.) | 34 (90%) | 4 (11%) | N.R. | |
| Sharma *et al.* 2020 [181] | RCT | Single center | Antegrade IMN | 24 | 42 (N.R.) | N.R. | N.R. | 12 (50%) | 4 (17%) | 8 (33%) | |
|  |  |  | Retrograde IMN | 19 | 44 (N.R.) | N.R. | N.R. | 9 (47%) | 6 (32%) | 4 (21%) | |
| Varghese *et al.* 2020 [182] | Prospective | Single center | MIPO | 5 | 43 (33-55) | 3 (60%) | 8 (3-12) | 1 (20%) | 4 (80%) | N.R. | |
| Wang *et al.* 2020 [183] | Retrospective | Single center | Antegrade IMN | 25 | 39 (18-56) | 16 (64%) | N.R. | 6 (24%) | 15 (60%) | 4 (16%) | |
|  |  |  | MIPO | 30 | 37 (18-56) | 17 (57%) | N.R. | 10 (33%) | 14 (47%) | 6 (20%) | |
| Wang *et al.* 2020 [184] | Prospective | Single center | Antegrade IMN | 26 | 46 (20-79) | 16 (62%) | 12 (N.R.) | 15 (58%) | 11 (42%) | N.R. | |
|  |  |  | ORPO | 30 | 45 (18-88) | 17 (57%) | 12 (N.R.) | 18 (60%) | 10 (33%) | 2 (7%) | |
| Yiğit *et al.* 2020 [185] | Retrospective | Single center | ORPO | 25 | 40 (20-65) | 16 (64%) | 46 (12-84) | 15 (60%) | 8 (32%) | 2 (8%) | |
| Zhang *et al.* 2020 [186] | Retrospective | Single center | Antegrade IMN | 34 | 43 (18-65) | 19 (56%) | 25 (N.R.) | 24 (71%) | 10 (29%) | N.R. | |
|  |  |  | ORPO | 46 | 41 (18-65) | 31 (67%) | 23 (N.R.) | 30 (65%) | 16 (35%) | N.R. | |
| Cannada *et al.* 2021 [187] | Prospective | Multicenter | Nonoperative Brace | 57 | 42 (N.R.) | 32 (56%) | 6 (N.R.) | 30 (53%) | 20 (35%) | 7 (12%) | |
|  |  |  | ORPO | 45 | 41 (N.R.) | 31 (69%) | 6 (N.R.) | 26 (57%) | 17 (38%) | 2 (5%) | |
| Capitani *et al.* 2021 [188] | Prospective | Single center | ORPO | 73 | 55 (N.R.) | 32 (44%) | 48 (N.R.) | 26 (36%) | 34 (47%) | 13 (18%) | |
| Furuhata *et al.* 2021 [189] | Retrospective | Single center | Antegrade IMN | 20 | 45 (15-89) | N.R. | N.R. | N.R. | N.R. | N.R. | |
|  |  |  | ORPO | 20 | 45 (15-89) | N.R. | N.R. | N.R. | N.R. | N.R. | |
| Huang *et al.* 2021 [190] | Retrospective | Single center | ORPO (anterolateral approach) | 86 | 45 (N.R.) | 44 (51%) | N.R. | 40 (47%) | 29 (34%) | 17 (20%) | |
|  |  |  | ORPO (posterior median approach) | 80 | 47 (N.R.) | 37 (46%) | N.R. | 33 (41%) | 32 (40%) | 15 (18%) | |
| Kumar *et al.* 2021 [191] | Retrospective | Single center | Nonoperative Brace | 40 | 36 (18-50) | 32 (80%) | 10 (N.R.) | 22 (55%) | 13 (33%) | 5 (13%) | |
|  |  |  | ORPO | 40 | 36 (18-50) | 30 (75%) | 11 (N.R.) | 20 (50%) | 14 (35%) | 6 (15%) | |
| Mohammed *et al.* 2021 [192] | Prospective | Multicenter | Antegrade IMN | 18 | 36 (21-54) | 7 (38%) | 5 (3-10) | 9 (50%) | 8 (44%) | 1 (6%) | |
|  |  |  | ORPO | 18 | 38 (21-54) | 12 (66%) | 5 (4-8) | 8 (44%) | 6 (33%) | 4 (22%) | |
| Patino *et al.* 2021 [193] | Retrospective | Single center | ORPO | 27 | 37 (N.R.) | 17 (63%) | 28 (N.R.) | 16 (59%) | 7 (26%) | 4 (15%) | |
| Rellán *et al.* 2021 [194] | Retrospective | Single center | MIPO | 35 | N.R. (47-75) | 10 (29%) | 8 (6-12) | 35 (100%) | N.R. | N.R. | |
|  |  |  | ORPO | 35 | N.R. (55-80) | 8 (23%) | 9 (6-18) | 35 (100%) | N.R. | N.R. | |

DCP, Dynamic Compression plate; IMN, Intramedullary nailing; LCP, Locking compression plate; MIPO, Minimally invasive plate

osteosynthesis; N.R., not reported; ORPO, Open reduction plate osteosynthesis; RCT, Randomized controlled trial.
