# Supplementary material for: Humeral shaft fracture: systematic review of non-operative and operative treatment
Source: Arch Orthop Trauma Surg. 2023 Apr 24;143(8):5035–54. doi: 10.1007/s00402-023-04836-8 (PMC10374687; doi:10.1007/s00402-023-04836-8)
Supplement: Supplementary file 4 — Supplementary file4 (DOCX 41 KB) [file 402_2023_4836_MOESM4_ESM.docx]

**Supplemental Table S2. MINORS score per study**

|  |  |  | A clearly stated aim | Inclusion of consecutive patients | Prospective collection of data | Endpoint appropriate to the aim of the study | Unbiased assessment of the study endpoint | Follow-up period appropriate to the aim of the study | Loss of follow up less than 5% | Prospective calculation of the study size | Additional criteria for comparative studies | | | |  | |  |
| --- | --- | --- | --- | --- | --- | --- | --- | --- | --- | --- | --- | --- | --- | --- | --- | --- | --- |
|  |  |  |  |  |  |  |  |  |  |  | An adequate control group | Contemporary group | Baseline equivalent of groups | Adequate statistical analysis |  | | |
| **Study** | **Study design** | **Comparative or noncomparative** |  |  |  |  |  |  |  |  |  |  |  |  | **Total score** | | |
|  |  |  |  |  |  |  |  |  |  |  |  |  |  |  |  | out of | |
| Sarmiento *et al*. 2000 [1] | Prospective | Noncomparative | 2 | 2 | 2 | 2 | 1 | 0 | 1 | 1 | N.A. | N.A. | N.A. | N.A. | 11 | 16 | |
| Chapman *et al*. 2000 [30] | RCT | Comparative | 2 | 2 | 2 | 2 | 1 | 2 | 0 | 1 | 2 | 2 | 2 | 1 | 19 | 24 | |
| McCormack *et al*. 2000 [31] | RCT | Comparative | 2 | 2 | 2 | 2 | 1 | 2 | 2 | 1 | 2 | 2 | 2 | 1 | 21 | 24 | |
| Paris *et al*. 2000 [32] | Retrospective | Noncomparative | 2 | 2 | 1 | 2 | 1 | 0 | 1 | 1 | N.A. | N.A. | N.A. | N.A. | 10 | 16 | |
| Tingstad *et al*. 2000 [33] | Retrospective | Noncomparative | 2 | 2 | 1 | 2 | 1 | 0 | 0 | 1 | N.A. | N.A. | N.A. | N.A. | 9 | 16 | |
| Blum *et al*. 2001 [34] | Prospective | Noncomparative | 2 | 2 | 2 | 2 | 1 | 2 | 0 | 1 | N.A. | N.A. | N.A. | N.A. | 12 | 16 | |
| Koch *et al*. 2002 [35] | Retrospective | Comparative | 2 | 2 | 1 | 2 | 1 | 0 | 0 | 1 | 1 | 2 | 1 | 1 | 14 | 24 | |
| Chaker *et al*. 2003 [36] | Retrospective | Comparative | 2 | 2 | 1 | 2 | 1 | 2 | 0 | 1 | 2 | 2 | 2 | 1 | 18 | 24 | |
| Kesemenli *et al*. 2003 [37] | Prospective | Comparative | 2 | 2 | 2 | 2 | 1 | 2 | 0 | 1 | 1 | 2 | 2 | 1 | 18 | 24 | |
| Ni *et al*. 2003 [38] | Retrospective | Noncomparative | 2 | 2 | 1 | 2 | 1 | 2 | 2 | 1 | N.A. | N.A. | N.A. | N.A. | 13 | 16 | |
| Fernandez *et al*. 2004 [39] | Prospective | Noncomparative | 2 | 2 | 2 | 1 | 1 | 2 | 1 | 1 | N.A. | N.A. | N.A. | N.A. | 12 | 16 | |
| Flinkkila *et al*. 2004 [40] | Retrospective | Comparative | 2 | 2 | 1 | 2 | 1 | 2 | 0 | 1 | 2 | 2 | 2 | 1 | 18 | 24 | |
| Livani *et al*. 2004 [41] | Prospective | Noncomparative | 2 | 2 | 2 | 1 | 1 | 2 | 2 | 1 | N.A. | N.A. | N.A. | N.A. | 13 | 16 | |
| Niall *et al*. 2004 [42] | Retrospective | Noncomparative | 2 | 2 | 1 | 2 | 1 | 0 | 1 | 1 | N.A. | N.A. | N.A. | N.A. | 10 | 16 | |
| Chao *et al*. 2005 [43] | Retrospective | Comparative | 2 | 2 | 1 | 2 | 1 | 2 | 0 | 1 | 2 | 2 | 2 | 1 | 18 | 24 | |
| Kirdemir *et al*. 2005 [44] | Retrospective | Noncomparative | 2 | 2 | 1 | 2 | 1 | 2 | 2 | 1 | N.A. | N.A. | N.A. | N.A. | 13 | 16 | |
| Toivanen *et al*. 2005 [45] | Retrospective | Noncomparative | 2 | 2 | 1 | 2 | 1 | 0 | 2 | 1 | N.A. | N.A. | N.A. | N.A. | 11 | 16 | |
| Rosenberg *et al*. 2006 [3] | Prospective | Noncomparative | 2 | 2 | 2 | 2 | 1 | 2 | 0 | 1 | N.A. | N.A. | N.A. | N.A. | 12 | 16 | |
| Apard *et al*. 2006 [46] | Prospective | Noncomparative | 2 | 1 | 2 | 2 | 1 | 2 | 2 | 1 | N.A. | N.A. | N.A. | N.A. | 13 | 16 | |
| Ekholm *et al*. 2006 [47] | Retrospective | Comparative | 2 | 2 | 1 | 2 | 1 | 0 | 0 | 1 | 2 | 2 | 2 | 2 | 17 | 24 | |
| Hernandez *et al*. 2006 [48] | Retrospective | Noncomparative | 2 | 2 | 1 | 2 | 1 | 1 | 1 | 1 | N.A. | N.A. | N.A. | N.A. | 11 | 16 | |
| Jawa *et al*. 2006 [49] | Retrospective | Comparative | 2 | 2 | 1 | 2 | 1 | 2 | 0 | 1 | 2 | 2 | 2 | 2 | 19 | 24 | |
| Martinez-Diaz *et al*. 2006 [50] | Retrospective | Noncomparative | 2 | 2 | 1 | 2 | 1 | 2 | 0 | 1 | N.A. | N.A. | N.A. | N.A. | 11 | 16 | |
| Pospula *et al*. 2006 [51] | Retrospective | Noncomparative | 2 | 2 | 1 | 1 | 1 | 0 | 2 | 1 | N.A. | N.A. | N.A. | N.A. | 10 | 16 | |
| Rochet *et al*. 2006 [52] | Retrospective | Noncomparative | 2 | 2 | 1 | 2 | 1 | 2 | 1 | 1 | N.A. | N.A. | N.A. | N.A. | 12 | 16 | |
| Rutgers *et al*. 2006 [53] | Retrospective | Noncomparative | 2 | 2 | 1 | 2 | 1 | 2 | 1 | 1 | N.A. | N.A. | N.A. | N.A. | 12 | 16 | |
| Cuny *et al*. 2007 [54] | Retrospective | Noncomparative | 2 | 2 | 1 | 2 | 1 | 2 | 1 | 1 | N.A. | N.A. | N.A. | N.A. | 12 | 16 | |
| Jiang *et al*. 2007 [55] | Prospective | Noncomparative | 2 | 2 | 2 | 2 | 1 | 2 | 1 | 1 | N.A. | N.A. | N.A. | N.A. | 13 | 16 | |
| Numbela *et al*. 2007 [56] | Prospective | Noncomparative | 2 | 1 | 2 | 2 | 1 | 2 | 0 | 1 | N.A. | N.A. | N.A. | N.A. | 11 | 16 | |
| Ozkurt *et al*. 2007 [57] | Prospective | Noncomparative | 2 | 1 | 2 | 2 | 1 | 2 | 0 | 1 | N.A. | N.A. | N.A. | N.A. | 11 | 16 | |
| Raghavendra *et al*. 2007 [58] | Prospective | Comparative | 2 | 2 | 2 | 2 | 1 | 0 | 2 | 1 | 2 | 2 | 2 | 2 | 20 | 24 | |
| Zhiquan *et al*. 2007 [59] | Prospective | Noncomparative | 2 | 1 | 2 | 2 | 1 | 2 | 2 | 1 | N.A. | N.A. | N.A. | N.A. | 13 | 16 | |
| Rommens *et al*. 2008 [22] | Retrospective | Noncomparative | 2 | 2 | 1 | 2 | 1 | 2 | 1 | 1 | N.A. | N.A. | N.A. | N.A. | 12 | 16 | |
| Cheng *et al*. 2008 [60] | RCT | Comparative | 2 | 2 | 2 | 2 | 1 | 2 | 2 | 2 | 2 | 2 | 2 | 2 | 23 | 24 | |
| Ekholm *et al*. 2008 [61] | Retrospective | Noncomparative | 2 | 2 | 1 | 2 | 1 | 2 | 1 | 1 | N.A. | N.A. | N.A. | N.A. | 12 | 16 | |
| Muckley *et al*. 2008 [62] | Prospective | Noncomparative | 2 | 2 | 2 | 2 | 1 | 2 | 2 | 1 | N.A. | N.A. | N.A. | N.A. | 14 | 16 | |
| An *et al*. 2009 [63] | Retrospective | Comparative | 2 | 2 | 1 | 2 | 1 | 2 | 1 | 1 | 2 | 2 | 2 | 2 | 20 | 24 | |
| Apivatthakakul *et al*. 2009 [64] | Retrospective | Noncomparative | 2 | 1 | 1 | 2 | 1 | 2 | 1 | 1 | N.A. | N.A. | N.A. | N.A. | 11 | 16 | |
| Ji *et al*. 2009 [65] | Retrospective | Noncomparative | 2 | 1 | 1 | 2 | 1 | 2 | 2 | 1 | N.A. | N.A. | N.A. | N.A. | 12 | 16 | |
| Li *et al*. 2009 [66] | RCT | Comparative | 2 | 2 | 2 | 2 | 2 | 2 | 2 | 1 | 2 | 2 | 2 | 2 | 23 | 24 | |
| Putti *et al*. 2009 [67] | RCT | Comparative | 2 | 2 | 2 | 2 | 1 | 2 | 0 | 1 | 2 | 2 | 2 | 2 | 20 | 24 | |
| Wang *et al*. 2009 [68] | Retrospective | Comparative | 2 | 2 | 1 | 2 | 1 | 2 | 0 | 1 | 2 | 2 | 2 | 2 | 19 | 24 | |
| An *et al*. 2010 [10] | Retrospective | Comparative | 2 | 2 | 1 | 2 | 1 | 2 | 2 | 1 | 2 | 2 | 2 | 2 | 21 | 24 | |
| Concha *et al*. 2010 [69] | Prospective | Noncomparative | 2 | 1 | 2 | 1 | 1 | 2 | 2 | 1 | N.A. | N.A. | N.A. | N.A. | 12 | 16 | |
| Denard *et al*. 2010 [70] | Retrospective | Comparative | 2 | 2 | 1 | 2 | 1 | 2 | 0 | 1 | 2 | 2 | 2 | 2 | 19 | 24 | |
| Denies *et al*. 2010 [71] | Retrospective | Comparative | 2 | 2 | 1 | 1 | 1 | 0 | 2 | 1 | 2 | 2 | 2 | 2 | 18 | 24 | |
| Kobayashi *et al*. 2010 [72] | Prospective | Noncomparative | 2 | 2 | 2 | 2 | 1 | 2 | 2 | 1 | N.A. | N.A. | N.A. | N.A. | 14 | 16 | |
| Singisetti *et al*. 2010 [73] | Prospective | Comparative | 2 | 2 | 2 | 2 | 1 | 2 | 1 | 1 | 2 | 2 | 2 | 0 | 19 | 24 | |
| Yin *et al*. 2010 [74] | Retrospective | Comparative | 2 | 2 | 1 | 2 | 1 | 2 | 2 | 1 | 2 | 2 | 2 | 2 | 21 | 24 | |
| Ziran *et al*. 2010 [75] | Retrospective | Noncomparative | 2 | 2 | 1 | 2 | 1 | 2 | 1 | 1 | N.A. | N.A. | N.A. | N.A. | 12 | 16 | |
| Algarin-Reyes *et al*. 2011 [76] | Retrospective | Comparative | 2 | 2 | 1 | 2 | 1 | 1 | 2 | 1 | 2 | 2 | 2 | 2 | 20 | 24 | |
| Grass *et al*. 2011 [77] | Retrospective | Noncomparative | 2 | 2 | 1 | 2 | 1 | 2 | 1 | 1 | N.A. | N.A. | N.A. | N.A. | 12 | 16 | |
| Kirin *et al*. 2011 [78] | Retrospective | Noncomparative | 2 | 2 | 1 | 2 | 1 | 0 | 0 | 1 | N.A. | N.A. | N.A. | N.A. | 9 | 16 | |
| Li *et al*. 2011 [79] | Retrospective | Comparative | 2 | 2 | 1 | 2 | 1 | 2 | 2 | 1 | 2 | 2 | 2 | 2 | 21 | 24 | |
| Lopez-Arevalo *et al*. 2011 [80] | Retrospective | Noncomparative | 2 | 2 | 1 | 2 | 1 | 0 | 2 | 1 | N.A. | N.A. | N.A. | N.A. | 11 | 16 | |
| Prasarn *et al*. 2011 [81] | Retrospective | Noncomparative | 2 | 1 | 1 | 2 | 1 | 2 | 1 | 1 | N.A. | N.A. | N.A. | N.A. | 11 | 16 | |
| Shetty *et al*. 2011 [82] | Prospective | Noncomparative | 2 | 2 | 2 | 2 | 2 | 2 | 2 | 1 | N.A. | N.A. | N.A. | N.A. | 15 | 16 | |
| Tsourvakas *et al*. 2011 [83] | Prospective | Noncomparative | 2 | 2 | 2 | 2 | 1 | 2 | 1 | 1 | N.A. | N.A. | N.A. | N.A. | 13 | 16 | |
| Van Middendorp *et al*. 2011 [84] | Prospective | Comparative | 2 | 1 | 2 | 2 | 1 | 2 | 2 | 2 | 2 | 2 | 2 | 2 | 22 | 24 | |
| Brunner *et al*. 2012 [85] | Retrospective | Noncomparative | 2 | 2 | 1 | 2 | 1 | 2 | 0 | 1 | N.A. | N.A. | N.A. | N.A. | 11 | 16 | |
| Firat *et al*. 2012 [86] | Retrospective | Comparative | 2 | 2 | 1 | 2 | 1 | 2 | 1 | 1 | 2 | 2 | 2 | 2 | 20 | 24 | |
| Iacobellis *et al*. 2012 [87] | Prospective | Noncomparative | 2 | 2 | 2 | 2 | 1 | 2 | 2 | 1 | N.A. | N.A. | N.A. | N.A. | 14 | 16 | |
| Idoine *et al*. 2012 [88] | Retrospective | Noncomparative | 2 | 2 | 1 | 2 | 1 | 2 | 1 | 1 | N.A. | N.A. | N.A. | N.A. | 12 | 16 | |
| Kulkarni *et al*. 2012 [89] | Retrospective | Comparative | 2 | 1 | 1 | 2 | 1 | 2 | 2 | 1 | 2 | 2 | 1 | 2 | 19 | 24 | |
| Kumar *et al*. 2012 [90] | Prospective | Comparative | 2 | 2 | 2 | 2 | 1 | 2 | 2 | 1 | 2 | 2 | 2 | 0 | 20 | 24 | |
| Malhan *et al*. 2012 [91] | Prospective | Noncomparative | 2 | 2 | 2 | 2 | 1 | 2 | 0 | 1 | N.A. | N.A. | N.A. | N.A. | 12 | 16 | |
| Oh *et al*. 2012 [92] | Retrospective | Comparative | 2 | 2 | 1 | 2 | 1 | 2 | 1 | 1 | 2 | 1 | 2 | 2 | 19 | 24 | |
| Pagonis *et al*. 2012 [93] | Retrospective | Noncomparative | 2 | 2 | 1 | 2 | 1 | 2 | 1 | 1 | N.A. | N.A. | N.A. | N.A. | 12 | 16 | |
| Shin *et al*. 2012 [94] | Prospective | Noncomparative | 2 | 1 | 2 | 2 | 1 | 2 | 2 | 1 | N.A. | N.A. | N.A. | N.A. | 13 | 16 | |
| Tan *et al*. 2012 [95] | Retrospective | Noncomparative | 2 | 1 | 1 | 2 | 1 | 2 | 0 | 1 | N.A. | N.A. | N.A. | N.A. | 10 | 16 | |
| Yang *et al*. 2012 [96] | Prospective | Noncomparative | 2 | 2 | 2 | 2 | 1 | 2 | 0 | 1 | N.A. | N.A. | N.A. | N.A. | 12 | 16 | |
| Zhou *et al*. 2012 [97] | Retrospective | Noncomparative | 2 | 1 | 1 | 2 | 1 | 2 | 1 | 1 | N.A. | N.A. | N.A. | N.A. | 11 | 16 | |
| Mahabier *et al*. 2013 [13] | Retrospective | Comparative | 2 | 2 | 1 | 2 | 1 | 0 | 0 | 1 | 2 | 2 | 2 | 2 | 17 | 24 | |
| Aydin *et al*. 2013 [98] | Retrospective | Noncomparative | 2 | 2 | 1 | 2 | 1 | 0 | 2 | 1 | N.A. | N.A. | N.A. | N.A. | 11 | 16 | |
| Biber *et al*. 2013 [99] | Retrospective | Comparative | 2 | 2 | 1 | 2 | 1 | 0 | 2 | 1 | 2 | 2 | 2 | 2 | 19 | 24 | |
| Boschi *et al*. 2013 [100] | Retrospective | Noncomparative | 2 | 2 | 1 | 2 | 1 | 2 | 2 | 1 | N.A. | N.A. | N.A. | N.A. | 13 | 16 | |
| Chen *et al*. 2013 [101] | Retrospective | Noncomparative | 2 | 2 | 1 | 2 | 1 | 2 | 1 | 1 | N.A. | N.A. | N.A. | N.A. | 12 | 16 | |
| Kapil Mani *et al*. 2013 [102] | Prospective | Noncomparative | 2 | 2 | 2 | 2 | 1 | 0 | 1 | 1 | N.A. | N.A. | N.A. | N.A. | 11 | 16 | |
| Lee *et al*. 2013 [103] | Prospective | Noncomparative | 2 | 2 | 2 | 2 | 1 | 2 | 0 | 1 | N.A. | N.A. | N.A. | N.A. | 12 | 16 | |
| Lee *et al*. 2013 [104] | Retrospective | Noncomparative | 2 | 2 | 1 | 2 | 1 | 2 | 1 | 1 | N.A. | N.A. | N.A. | N.A. | 12 | 16 | |
| Lian *et al*. 2013 [105] | RCT | Comparative | 2 | 2 | 2 | 2 | 1 | 2 | 1 | 1 | 2 | 2 | 2 | 2 | 21 | 24 | |
| Sharaby *et al*. 2013 [106] | Prospective | Noncomparative | 2 | 2 | 2 | 2 | 1 | 2 | 2 | 1 | N.A. | N.A. | N.A. | N.A. | 14 | 16 | |
| Shen *et al*. 2013 [107] | Retrospective | Comparative | 2 | 2 | 1 | 2 | 1 | 2 | 1 | 1 | 2 | 2 | 2 | 2 | 20 | 24 | |
| Tyllianakis *et al*. 2013 [108] | Retrospective | Noncomparative | 2 | 2 | 1 | 2 | 1 | 2 | 1 | 1 | N.A. | N.A. | N.A. | N.A. | 12 | 16 | |
| Verdano *et al*. 2013 [109] | Retrospective | Noncomparative | 2 | 2 | 1 | 2 | 1 | 2 | 1 | 1 | N.A. | N.A. | N.A. | N.A. | 12 | 16 | |
| Wang *et al*. 2013 [110] | RCT | Comparative | 2 | 2 | 2 | 2 | 1 | 2 | 1 | 1 | 1 | 2 | 2 | 2 | 20 | 24 | |
| Yi *et al*. 2013 [111] | Prospective | Noncomparative | 2 | 2 | 2 | 2 | 1 | 2 | 1 | 1 | N.A. | N.A. | N.A. | N.A. | 13 | 16 | |
| Yin *et al*. 2013 [112] | Retrospective | Noncomparative | 2 | 2 | 1 | 2 | 1 | 2 | 1 | 1 | N.A. | N.A. | N.A. | N.A. | 12 | 16 | |
| Baltov *et al*. 2014 [5] | Retrospective | Noncomparative | 2 | 2 | 1 | 2 | 1 | 2 | 2 | 1 | N.A. | N.A. | N.A. | N.A. | 13 | 16 | |
| Balam *et al*. 2014 [113] | Prospective | Noncomparative | 2 | 2 | 2 | 2 | 1 | 2 | 2 | 1 | N.A. | N.A. | N.A. | N.A. | 14 | 16 | |
| Benegas *et al*. 2014 [114] | RCT | Comparative | 2 | 2 | 2 | 2 | 1 | 2 | 2 | 2 | 2 | 2 | 2 | 2 | 23 | 24 | |
| Huri *et al*. 2014 [115] | Retrospective | Noncomparative | 2 | 2 | 1 | 2 | 1 | 2 | 0 | 1 | N.A. | N.A. | N.A. | N.A. | 11 | 16 | |
| Neuhaus *et al*. 2014 [116] | Retrospective | Noncomparative | 2 | 2 | 1 | 2 | 1 | 2 | 2 | 1 | N.A. | N.A. | N.A. | N.A. | 13 | 16 | |
| Radulescu *et al*. 2014 [117] | Prospective | Comparative | 2 | 2 | 2 | 2 | 1 | 0 | 0 | 1 | 2 | 2 | 2 | 1 | 17 | 24 | |
| Singh *et al*. 2014 [118] | Retrospective | Comparative | 2 | 2 | 1 | 2 | 1 | 2 | 2 | 1 | 2 | 2 | 2 | 2 | 21 | 24 | |
| Wali *et al*. 2014 [119] | RCT | Comparative | 2 | 2 | 2 | 2 | 1 | 0 | 0 | 1 | 2 | 2 | 2 | 1 | 17 | 24 | |
| Wang *et al*. 2014 [120] | Prospective | Comparative | 2 | 2 | 2 | 2 | 1 | 2 | 1 | 1 | 2 | 2 | 2 | 2 | 21 | 24 | |
| Yin *et al*. 2014 [121] | Retrospective | Comparative | 2 | 2 | 1 | 2 | 1 | 2 | 2 | 1 | 2 | 2 | 2 | 2 | 21 | 24 | |
| Zogaib *et al*. 2014 [122] | Retrospective | Noncomparative | 2 | 2 | 1 | 2 | 1 | 2 | 2 | 1 | N.A. | N.A. | N.A. | N.A. | 13 | 16 | |
| Zogbi *et al*. 2014 [123] | Retrospective | Noncomparative | 2 | 2 | 1 | 2 | 1 | 2 | 1 | 1 | N.A. | N.A. | N.A. | N.A. | 12 | 16 | |
| Chen *et al*. 2015 [11] | Retrospective | Comparative | 2 | 2 | 1 | 2 | 1 | 2 | 1 | 2 | 2 | 2 | 2 | 2 | 21 | 24 | |
| Ali *et al*. 2015 [124] | Retrospective | Noncomparative | 2 | 2 | 1 | 2 | 1 | 0 | 1 | 1 | N.A. | N.A. | N.A. | N.A. | 10 | 16 | |
| Campochiaro *et al*. 2015 [125] | Retrospective | Noncomparative | 2 | 2 | 1 | 2 | 1 | 2 | 1 | 1 | N.A. | N.A. | N.A. | N.A. | 12 | 16 | |
| Ebrahimpour *et al*. 2015 [126] | Prospective | Noncomparative | 2 | 2 | 2 | 2 | 1 | 2 | 2 | 1 | N.A. | N.A. | N.A. | N.A. | 14 | 16 | |
| Esmailiejah *et al*. 2015 [127] | Prospective | Comparative | 2 | 2 | 2 | 2 | 1 | 0 | 2 | 1 | 2 | 2 | 2 | 2 | 20 | 24 | |
| Fan *et al*. 2015 [128] | RCT | Comparative | 2 | 2 | 2 | 2 | 1 | 0 | 0 | 2 | 2 | 2 | 2 | 2 | 19 | 24 | |
| Feng *et al*. 2015 [129] | RCT | Comparative | 2 | 2 | 2 | 2 | 1 | 2 | 1 | 1 | 2 | 2 | 2 | 2 | 21 | 24 | |
| Gallucci *et al*. 2015 [130] | Retrospective | Noncomparative | 2 | 2 | 1 | 2 | 1 | 2 | 2 | 1 | N.A. | N.A. | N.A. | N.A. | 13 | 16 | |
| Hadhoud *et al*. 2015 [131] | RCT | Comparative | 2 | 2 | 2 | 2 | 1 | 2 | 0 | 1 | 2 | 2 | 2 | 2 | 20 | 24 | |
| Kim *et al*. 2015 [132] | Retrospective | Noncomparative | 2 | 2 | 1 | 2 | 1 | 2 | 1 | 1 | N.A. | N.A. | N.A. | N.A. | 12 | 16 | |
| Koca *et al*. 2015 [133] | Retrospective | Noncomparative | 2 | 2 | 1 | 2 | 1 | 2 | 0 | 1 | N.A. | N.A. | N.A. | N.A. | 11 | 16 | |
| Kumar *et al*. 2015 [134] | Prospective | Noncomparative | 2 | 2 | 2 | 2 | 1 | 2 | 0 | 1 | N.A. | N.A. | N.A. | N.A. | 12 | 16 | |
| Modi *et al*. 2015 [135] | RCT | Comparative | 2 | 2 | 2 | 2 | 1 | 2 | 1 | 1 | 2 | 2 | 2 | 0 | 19 | 24 | |
| Patino *et al*. 2015 [136] | Retrospective | Noncomparative | 2 | 2 | 1 | 2 | 1 | 2 | 1 | 1 | N.A. | N.A. | N.A. | N.A. | 12 | 16 | |
| Reddy *et al*. 2015 [137] | Prospective | Noncomparative | 2 | 2 | 2 | 2 | 1 | 1 | 0 | 1 | N.A. | N.A. | N.A. | N.A. | 11 | 16 | |
| Sahu *et al*. 2015 [138] | Prospective | Noncomparative | 2 | 2 | 2 | 2 | 1 | 0 | 2 | 1 | N.A. | N.A. | N.A. | N.A. | 12 | 16 | |
| Sanjeevaiah *et al*. 2015 [139] | Prospective | Noncomparative | 2 | 2 | 2 | 2 | 1 | 2 | 2 | 1 | N.A. | N.A. | N.A. | N.A. | 14 | 16 | |
| Shields *et al*. 2015 [140] | Retrospective | Noncomparative | 2 | 2 | 1 | 2 | 1 | 2 | 1 | 1 | N.A. | N.A. | N.A. | N.A. | 12 | 16 | |
| Singhal *et al*. 2015 [141] | Retrospective | Noncomparative | 2 | 2 | 1 | 2 | 1 | 1 | 2 | 1 | N.A. | N.A. | N.A. | N.A. | 12 | 16 | |
| Srinivas *et al*. 2015 [142] | Prospective | Noncomparative | 2 | 2 | 2 | 2 | 1 | 1 | 0 | 1 | N.A. | N.A. | N.A. | N.A. | 11 | 16 | |
| Wang *et al*. 2015 [143] | Prospective | Comparative | 2 | 2 | 2 | 2 | 1 | 2 | 1 | 2 | 2 | 2 | 2 | 2 | 22 | 24 | |
| Abril Goano *et al*. 2016 [144] | Prospective | Noncomparative | 2 | 2 | 2 | 2 | 1 | 1 | 0 | 1 | N.A. | N.A. | N.A. | N.A. | 11 | 16 | |
| Anand Kumar *et al*. 2016 [145] | Prospective | Noncomparative | 2 | 2 | 2 | 2 | 1 | 2 | 2 | 1 | N.A. | N.A. | N.A. | N.A. | 14 | 16 | |
| Gang *et al*. 2016 [146] | Retrospective | Comparative | 2 | 2 | 1 | 2 | 1 | 2 | 0 | 1 | 2 | 2 | 1 | 0 | 16 | 24 | |
| Guzmán-Guevara *et al*. 2016 [147] | Prospective | Comparative | 2 | 2 | 2 | 2 | 1 | 2 | 2 | 1 | 2 | 2 | 2 | 1 | 21 | 24 | |
| Karunanithi *et al*. 2016 [148] | Prospective | Noncomparative | 2 | 2 | 2 | 2 | 1 | 2 | 0 | 1 | N.A. | N.A. | N.A. | N.A. | 12 | 16 | |
| Kumar *et al*. 2016 [149] | Prospective | Comparative | 2 | 2 | 2 | 2 | 1 | 0 | 0 | 1 | 2 | 2 | 2 | 2 | 18 | 24 | |
| Lee *et al*. 2016 [150] | Retrospective | Noncomparative | 2 | 2 | 1 | 2 | 1 | 2 | 1 | 1 | N.A. | N.A. | N.A. | N.A. | 12 | 16 | |
| Lee *et al*. 2016 [151] | Retrospective | Comparative | 2 | 2 | 1 | 2 | 1 | 2 | 2 | 1 | 2 | 2 | 2 | 2 | 21 | 24 | |
| Lu *et al*. 2016 [152] | Retrospective | Comparative | 2 | 2 | 1 | 2 | 1 | 2 | 0 | 1 | 2 | 2 | 2 | 1 | 18 | 24 | |
| Mahajan *et al*. 2016 [153] | Prospective | Noncomparative | 2 | 2 | 2 | 2 | 1 | 2 | 1 | 1 | N.A. | N.A. | N.A. | N.A. | 13 | 16 | |
| Mehmood *et al*. 2016 [154] | RCT | Comparative | 2 | 2 | 2 | 2 | 1 | 1 | 0 | 1 | 2 | 2 | 2 | 2 | 19 | 24 | |
| Wahed *et al*. 2016 [155] | Prospective | Noncomparative | 2 | 2 | 2 | 2 | 1 | 2 | 0 | 1 | N.A. | N.A. | N.A. | N.A. | 12 | 16 | |
| Bisaccia *et al*. 2017 [156] | Retrospective | Comparative | 2 | 2 | 1 | 2 | 1 | 2 | 0 | 1 | 2 | 2 | 2 | 2 | 19 | 24 | |
| Dielwart *et al*. 2017 [157] | Retrospective | Comparative | 2 | 2 | 1 | 2 | 1 | 2 | 1 | 1 | 2 | 2 | 2 | 2 | 20 | 24 | |
| Duygun *et al*. 2017 [158] | Prospective | Noncomparative | 2 | 2 | 2 | 2 | 1 | 2 | 2 | 1 | N.A. | N.A. | N.A. | N.A. | 14 | 16 | |
| Harkin *et al*. 2017 [159] | Retrospective | Comparative | 2 | 2 | 1 | 2 | 1 | 1 | 1 | 1 | 2 | 2 | 2 | 2 | 19 | 24 | |
| Ko *et al*. 2017 [160] | Retrospective | Comparative | 2 | 1 | 1 | 2 | 1 | 2 | 0 | 1 | 2 | 2 | 2 | 2 | 18 | 24 | |
| Matsunaga *et al*. 2017 [161] | RCT | Comparative | 2 | 2 | 2 | 2 | 2 | 2 | 1 | 2 | 2 | 2 | 2 | 2 | 23 | 24 | |
| ShengWei *et al*. 2017 [162] | Retrospective | Comparative | 2 | 2 | 1 | 2 | 1 | 2 | 0 | 1 | 2 | 2 | 2 | 2 | 19 | 24 | |
| Crespo *et al*. 2018 [163] | Retrospective | Noncomparative | 2 | 2 | 1 | 2 | 1 | 2 | 2 | 1 | N.A. | N.A. | N.A. | N.A. | 13 | 16 | |
| Goncalves *et al*. 2018 [164] | Retrospective | Comparative | 2 | 2 | 1 | 2 | 1 | 2 | 1 | 1 | 2 | 2 | 1 | 2 | 19 | 24 | |
| Ferrara *et al*. 2019 [165] | Retrospective | Noncomparative | 2 | 2 | 1 | 2 | 1 | 2 | 1 | 1 | N.A. | N.A. | N.A. | N.A. | 12 | 16 | |
| Hosseini Khameneh *et al*. 2019 [166] | RCT | Comparative | 2 | 2 | 2 | 2 | 1 | 2 | 0 | 1 | 2 | 2 | 2 | 2 | 20 | 24 | |
| Mehraj *et al*. 2019 [167] | Prospective | Noncomparative | 2 | 2 | 2 | 2 | 1 | 2 | 0 | 1 | N.A. | N.A. | N.A. | N.A. | 12 | 16 | |
| Pooja *et al*. 2019 [168] | RCT | Comparative | 2 | 2 | 2 | 2 | 2 | 2 | 2 | 1 | 2 | 2 | 2 | 2 | 23 | 24 | |
| Seo *et al*. 2019 [169] | Prospective | Comparative | 2 | 2 | 2 | 2 | 1 | 2 | 2 | 1 | 2 | 2 | 2 | 2 | 22 | 24 | |
| Vidovic *et al*. 2019 [170] | Retrospective | Noncomparative | 2 | 2 | 1 | 2 | 1 | 1 | 0 | 1 | N.A. | N.A. | N.A. | N.A. | 10 | 16 | |
| Wang *et al*. 2019 [171] | Retrospective | Comparative | 2 | 2 | 1 | 2 | 2 | 2 | 0 | 1 | 2 | 2 | 2 | 2 | 20 | 24 | |
| Yuan *et al*. 2019 [172] | Retrospective | Comparative | 2 | 2 | 1 | 2 | 1 | 2 | 0 | 1 | 2 | 2 | 2 | 2 | 19 | 24 | |
| Li *et al*. 2020 [7] | Retrospective | Comparative | 2 | 2 | 1 | 2 | 1 | 0 | 0 | 1 | 2 | 2 | 2 | 2 | 17 | 24 | |
| Akalin *et al*. 2020 [173] | RCT | Comparative | 2 | 2 | 2 | 2 | 1 | 2 | 2 | 1 | 2 | 2 | 2 | 2 | 22 | 24 | |
| Desai *et al*. 2020 [174] | RCT | Comparative | 2 | 2 | 2 | 2 | 1 | 1 | 0 | 1 | 2 | 2 | 2 | 2 | 19 | 24 | |
| Hendy *et al*. 2020 [175] | Retrospective | Comparative | 2 | 2 | 1 | 2 | 1 | 2 | 1 | 1 | 2 | 2 | 2 | 2 | 20 | 24 | |
| Huichao *et al*. 2020 [176] | Retrospective | Comparative | 2 | 2 | 1 | 2 | 1 | 2 | 0 | 1 | 2 | 2 | 2 | 1 | 18 | 24 | |
| Hussain *et al*. 2020 [177] | RCT | Comparative | 2 | 2 | 2 | 2 | 1 | 1 | 0 | 1 | 2 | 2 | 2 | 1 | 18 | 24 | |
| Omrani *et al*. 2020 [178] | RCT | Comparative | 2 | 2 | 2 | 2 | 1 | 2 | 0 | 1 | 2 | 2 | 2 | 2 | 20 | 24 | |
| Rai *et al*. 2020 [179] | Prospective | Noncomparative | 2 | 2 | 2 | 2 | 1 | 2 | 0 | 1 | N.A. | N.A. | N.A. | N.A. | 12 | 16 | |
| Rämö *et al*. 2020 [180] | RCT | Comparative | 2 | 2 | 2 | 2 | 1 | 2 | 1 | 2 | 2 | 2 | 2 | 2 | 22 | 24 | |
| Sharma *et al*. 2020 [181] | RCT | Comparative | 2 | 2 | 2 | 2 | 1 | 2 | 2 | 1 | 2 | 2 | 2 | 2 | 22 | 24 | |
| Varghese *et al*. 2020 [182] | Prospective | Noncomparative | 2 | 2 | 2 | 2 | 1 | 2 | 2 | 1 | N.A. | N.A. | N.A. | N.A. | 14 | 16 | |
| Wang *et al*. 2020 [183] | Retrospective | Comparative | 2 | 2 | 1 | 2 | 1 | 2 | 0 | 1 | 2 | 2 | 2 | 2 | 19 | 24 | |
| Wang *et al*. 2020 [184] | Prospective | Comparative | 2 | 2 | 2 | 2 | 1 | 2 | 1 | 1 | 2 | 2 | 2 | 2 | 21 | 24 | |
| Yiğit *et al*. 2020 [185] | Retrospective | Noncomparative | 2 | 2 | 1 | 2 | 1 | 2 | 0 | 1 | N.A. | N.A. | N.A. | N.A. | 11 | 16 | |
| Zhang *et al*. 2020 [186] | Retrospective | Comparative | 2 | 2 | 1 | 2 | 1 | 2 | 0 | 1 | 2 | 2 | 2 | 2 | 19 | 24 | |
| Cannada *et al*. 2021 [187] | Prospective | Comparative | 2 | 2 | 2 | 2 | 1 | 2 | 1 | 2 | 2 | 2 | 2 | 2 | 22 | 24 | |
| Capitani *et al*. 2021 [188] | Prospective | Noncomparative | 2 | 2 | 2 | 2 | 1 | 2 | 2 | 1 | N.A. | N.A. | N.A. | N.A. | 14 | 16 | |
| Furuhata *et al*. 2021 [189] | Retrospective | Comparative | 2 | 2 | 1 | 2 | 1 | 1 | 0 | 1 | 2 | 2 | 2 | 2 | 18 | 24 | |
| Huang *et al*. 2021 [190] | Retrospective | Comparative | 2 | 2 | 1 | 2 | 1 | 2 | 0 | 1 | 2 | 2 | 2 | 2 | 19 | 24 | |
| Kumar *et al*. 2021 [191] | Prospective | Noncomparative | 2 | 2 | 2 | 2 | 1 | 1 | 0 | 1 | N.A. | N.A. | N.A. | N.A. | 11 | 16 | |
| Mohammed *et al*. 2021 [192] | Prospective | Comparative | 2 | 2 | 2 | 2 | 1 | 1 | 0 | 1 | 2 | 2 | 2 | 1 | 18 | 24 | |
| Patino *et al*. 2021 [193] | Retrospective | Comparative | 2 | 2 | 1 | 2 | 1 | 2 | 0 | 1 | 2 | 2 | 2 | 2 | 19 | 24 | |
| Rellán *et al*. 2021 [194] | Retrospective | Comparative | 2 | 2 | 1 | 2 | 1 | 2 | 0 | 1 | 2 | 2 | 2 | 2 | 19 | 24 | |

N.A., not applicable; RCT, Randomized controlled trial.
