# Supplementary material for: Humeral shaft fracture: systematic review of non-operative and operative treatment
Source: Arch Orthop Trauma Surg. 2023 Apr 24;143(8):5035–54. doi: 10.1007/s00402-023-04836-8 (PMC10374687; doi:10.1007/s00402-023-04836-8)
Supplement: Supplementary file 5 — Supplementary file5 (DOCX 25 KB) [file 402_2023_4836_MOESM5_ESM.docx]

**Supplemental Table S3. Complication rates of (treatment of) a humeral shaft fracture per treatment group**

|  | **Treatment** | **Study arms** | **Population** | **Cases** | **Heterogeneity** | | **Pooled value** |
| --- | --- | --- | --- | --- | --- | --- | --- |
|  |  | **N** | **N** | **N** | **Cochran’s Q**  **(p-value)** | **I^2^ (%)**  **(95% CI)** | **(%)**  **(95% CI)** |
| **Shoulder dysfunction^a^**  [30, 32, 34, 50, 66, 67, 73, 75, 84, 86, 90, 114, 119, 136-138, 141, 147, 149, 165, 174, 177, 181, 183, 184, 191] | **Nonoperative** | 3 | 122 | 9 | 17 (<0.001) | 88 (67-96) | 9 (0-29) |
|  | **IMN** | 22 | 693 | 74 | 43 (<0.001) | 52 (21-70) | 11 (8-15) |
|  | **Antegrade** | 19 | 563 | 70 | 28 (0.061) | 37 (0-64) | 13 (10-16) |
|  | **Retrograde** | 2 | 46 | 2 | 2 (0.196) | 40 (0-0) | 5 (1-15) |
|  | **Plate** | 15 | 560 | 34 | 24 (0.048) | 41 (0-68) | 5 (3-8) |
|  | **ORPO** | 12 | 477 | 30 | 20 (0.048) | 44 (0-72) | 5 (3-9) |
|  | **MIPO** | 3 | 83 | 4 | 4 (0.148) | 48 (0-85) | 5 (1-12) |
| **Nail protrusion^b^**  [5, 31, 38, 43, 46, 48, 50, 52, 54, 58, 67, 84, 86, 87, 105, 108, 158 | **Nonoperative** | N.A. | N.A. | N.A. | N.A. | N.A. | N.A. |
|  | **IMN** | 17 | 666 | 61 | 40 (<0.001) | 60 (32-77) | 10 (6-14) |
|  | **Antegrade** | 11 | 404 | 31 | 27 (0.002) | 63 (30-81) | 9 (5-14) |
|  | **Retrograde** | 2 | 83 | 4 | 0 (0.866) | 0 (0-0) | 6 (2-13) |
|  | **Plate** | N.A. | N.A. | N.A. | N.A. | N.A. | N.A. |
|  | **ORPO** | N.A. | N.A. | N.A. | N.A. | N.A. | N.A. |
|  | **MIPO** | N.A. | N.A. | N.A. | N.A. | N.A. | N.A. |
| **Subacromial impingement^c^**  [37, 50, 58, 60, 86, 90, 97, 112, 119, 135-137, 158, 162, 183, 186] | **Nonoperative** | N.A. | N.A. | N.A. | N.A. | N.A. | N.A. |
|  | **IMN** | 17 | 500 | 67 | 33 (0.007) | 52 (16-72) | 14 (10-19) |
|  | **Antegrade** | 15 | 432 | 55 | 29 (0.012) | 51 (11-73) | 13 (9-18) |
|  | **Retrograde** | 0 | N.A. | N.A. | N.A. | N.A. | N.A. |
|  | **Plate** | 10 | 368 | 5 | 8 (0.507) | 0 (0-59) | 2 (1-3) |
|  | **ORPO** | 8 | 264 | 1 | 3 (0.903) | 0 (0-20) | 1 (0-3) |
|  | **MIPO** | 2 | 104 | 4 | 2 (0.142) | 54 (0-89) | 4 (1-10) |
| **(Sub)cutaneous^d^**  [35, 49, 53, 57, 84, 86, 140, 161, 180] | **Nonoperative** | 9 | 347 | 20 | 14 (0.087) | 42 (0-73) | 6 (4-9) |
|  | **IMN** | 1 | N.A. | N.A. | N.A. | N.A. | N.A. |
|  | **Antegrade** | 1 | N.A. | N.A. | N.A. | N.A. | N.A. |
|  | **Retrograde** | 0 | N.A. | N.A. | N.A. | N.A. | N.A. |
|  | **Plate** | 10 | 387 | 8 | 11 (0.310) | 14 (0-56) | 2 (1-4) |
|  | **ORPO** | 7 | 255 | 3 | 6 (0.374) | 7 (0-73) | 1 (0-4) |
|  | **MIPO** | 3 | 132 | 5 | 1 (0.743) | 0 (0-89) | 5 (2-10) |

^a^ Shoulder dysfunction was defined as experiencing pain or limited range of motion of the shoulder.

^b^ Nail protrusion was defined as migration and subsequent protrusion of the intramedullary nail.

^c^ Subacromial impingement was defined as irritation of the rotator cuff muscles in the subacromial space.

^d^ (Sub)cutaneous problems included but were not limited to bursitis, cellulitis, granuloma’s, hypertrophic scarring of the wound, and skin irritation, macerations, or abrasions due to prolonged contact with the brace*.*

95% CI, 95% Confidence interval; IMN, Intramedullary nailing; MIPO, Minimally invasive plate osteosynthesis; N.A., not applicable; ORPO, Open reduction plate osteosynthesis.
