# Supplementary material for: Humeral shaft fracture: systematic review of non-operative and operative treatment
Source: Arch Orthop Trauma Surg. 2023 Apr 24;143(8):5035–54. doi: 10.1007/s00402-023-04836-8 (PMC10374687; doi:10.1007/s00402-023-04836-8)
Supplement: Supplementary file 6 — Supplementary file6 (DOCX 24 KB) [file 402_2023_4836_MOESM6_ESM.docx]

**Supplemental Table S4. Functional outcome scores after treatment of a humeral shaft fracture per treatment group**

| **Instrument** | **Treatment** | **Study arms** | **Population** | **Heterogeneity** | | **Pooled value** |
| --- | --- | --- | --- | --- | --- | --- |
|  |  | **N** | **N** | **Cochran’s Q**  **(p-value)** | **I^2^ (%)**  **(95% CI)** | **(points)**  **(95% CI)** |
| **ASES score^a^**  [7, 110, 115, 128, 143, 160, 183, 184] | **Nonoperative** | 0 | N.A. | N.A. | N.A. | N.A. |
|  | **IMN** | 5 | 125 | 13 (0.001) | 70 (24-88) | 91 (90-93) |
|  | **Antegrade** | 5 | 125 | 13 (0.001) | 70 (24-88) | 91 (90-93) |
|  | **Retrograde** | 0 | N.A. | N.A. | N.A. | N.A. |
|  | **Plate** | 10 | 242 | 158 (<0.001) | 94 (91-96) | 92 (91-94) |
|  | **ORPO** | 7 | 163 | 127 (<0.001) | 95 (92-97) | 93 (92-95) |
|  | **MIPO** | 3 | 79 | 30 (<0.001) | 93 (84-97) | 91 (89-94) |
| **MEPI^b^**  [10, 11, 60, 66, 79, 107, 115, 118, 121, 123, 127, 131, 146, 151, 153, 172, 183] | **Nonoperative** | 0 | N.A. | N.A. | N.A. | N.A. |
|  | **IMN** | 6 | 487 | 949 (<0.001) | 99 (99-100) | 95 (92-98) |
|  | **Antegrade** | 5 | 442 | 928 (<0.001) | 100 (99-100) | 95 (91-98) |
|  | **Retrograde** | 1 | N.A. | N.A. | N.A. | N.A. |
|  | **Plate** | 23 | 914 | 2,087 (<0.001) | 99 (99-99) | 94 (93-96) |
|  | **ORPO** | 14 | 520 | 369 (<0.001) | 96 (95-97) | 95 (94-96) |
|  | **MIPO** | 9 | 394 | 453 (<0.001) | 98 (98-99) | 95 (91-98) |
| **Pain (VAS)^c^**  [161, 183, 184, 192] | **Nonoperative** | 0 | N.A. | N.A. | N.A. | N.A. |
|  | **IMN** | 3 | 69 | 119 (<0.001) | 98 (97-99) | 2 (0-4) |
|  | **Antegrade** | 3 | 69 | 119 (<0.001) | 98 (97-99) | 2 (0-4) |
|  | **Retrograde** | 0 | N.A. | N.A. | N.A. | N.A. |
|  | **Plate** | 3 | 107 | 485 (<0.001) | 100 (99-100) | 2 (0-5) |
|  | **ORPO** | 2 | 77 | 5 (0.025) | 80 (14-95) | 1 (0-1) |
|  | **MIPO** | 1 | 30 | N.A. | N.A. | N.A. |

^a^ The American Shoulder and Elbow Surgeons (ASES) score ranges from 0 to100 points, with a higher score representing better outcome [200].

^b^ The Mayo Elbow Performance Index (MEPI) ranges from 5 to 100 points, with a higher score representing better outcome [201].

^c^ The level of pain was measured with a Visual Analog Scale (VAS), ranging from 0 to 10 points, with a higher score representing more pain.

95% CI, 95% Confidence interval; ASES, American Shoulder and Elbow Surgeons; IMN, Intramedullary nailing; MEPI, Mayo Elbow Performance Index; MIPO, Minimally invasive plate osteosynthesis; N.A., not applicable; ORPO, Open reduction plate osteosynthesis; VAS, Visual Analog Scale.
